# Uterine infusion strategies for infertile patients with recurrent implantation failure: a systematic review and network meta-analysis

**DOI:** 10.1186/s12958-024-01221-x

**Published:** 2024-04-16

**Authors:** Qin Xie, Xiaozhen Quan, Yanli Lan, Xuezhou Yang

**Affiliations:** Department of Obstetrics and Gynecology, Xiangyang Central Hospital, The Affiliated Hospital of Hubei University of Arts and Science, Xiangyang, Hubei 441021 P. R. China

**Keywords:** Repeated implantation failure (RIF), Intra-uterine infusion, Platelet-rich plasma (PRP), Granulocyte Colony-Stimulating Factor (G-CSF), Peripheral Blood Mononuclear Cell (PBMC)

## Abstract

**Background:**

Intra-uterine infusion treatments were reported to be beneficial to embryo implantation and pregnancy outcomes, and considered as potential therapies for infertile patients with recurrent implantation failure (RIF). Nevertheless, their efficiencies were controversial and there lack of consensus on which intrauterine treatment is the most effective.

**Methods:**

All prospective trials (in Chinese or English) were searched in Databases PubMed, Cochrane, Web of Science, and CNKI from July 2013 to July 2023. We included studies that investigated various uterine infusions, including chorionic gonadotropin, granulocyte colony-stimulating factor, monocytes, platelet-rich plasma, etc. during IVF treatment and reported subsequent pregnancy outcomes.

**Results:**

We finally included 56 researches, including 40 randomized controlled trials, 14 non-randomized controlled trials, and 3 prospective cohort studies. This study included a total of 11 uterine perfusion methods: Placebo, Human Chorionic Gonadotropin (HCG), Granulocyte Colony-Stimulating Factor (G-CSF), platelet-rich plasma (PRP), Peripheral Blood Mononuclear Cell (PBMC), Growth hormone (GH), dexamethasone (DEX), Embryo culture supernatant (ESC), PRP combined with G-CSF (PRP + G-CSF), RPR combined with subcutaneous injection of G-CSF (RPR + G-CSFsc), G-CSF combined with subcutaneous injection of AXaIU (G-CSF + AXaIUsc). Intrauterine infusion of HCG, PBMC, G-CSF, and PRP significantly improves pregnancy outcomes in patients with repeated implantation failure compared with blank controls or placebo, and PRP improved the clinical pregnancy and live birth most. GH and ESC infusion might improve the pregnancy outcomes, but uterine infusion of DEX was shown with high miscarriage. The combination therapy did not show a significant advantage over the mono-therapy.

**Conclusions:**

Intrauterine infusion of HCG, PBMC, G-CSF, and PRP are promising strategies for improving pregnancy outcomes for infertile patients with recurrent implantation failure. Among these treatments, PRP may be the best. More researches are required to explore the effect of drug combinations and less commonly used drugs as well.

**Trial registration:**

Our study was registered in PROSPERO and the ID was CRD42023467188.

**Supplementary Information:**

The online version contains supplementary material available at 10.1186/s12958-024-01221-x.

## Synopsis

Intrauterine infusion seems a promising strategy for improving the pregnancy outcomes for infertile patients with recurrent implantation failure.

## Introduction

Assisted reproductive technology (ART) has been widely used since the first baby was born in 1778s. Nowadays, more than 8 million babies are born with the help of ART worldwide [1]. However, live birth rates of this treatment need to be improved, for the clinical pregnancy rate is between 30 and 40% [2], and the live birth rate is estimated to be between 20 and 29% [3, 4]. Implantation failure is one of the tough bottlenecks of ART success [5, 6].

Successful embryo implantation is a complex process, requiring a competent embryo, a receptive endometrium, and an appropriate dialogue [7]. The competence of the embryo is influenced by various factors, such as age, environmental pollutant exposure, and unhealthy lifestyle. Endometrial receptivity can be altered by endometrial blood supply, immune state, the structural uterine malformation (such as polyps and adhesions). An appropriate dialogue includes embryo location, adhesion, and intrusion. Synchronous development between embryo and endometrium was reported to be involved. In infertile patients with recurrent implantation failure, despite the transfer of good-quality embryos, the implantation was aborted, indicating the significant difference in the endometrial receptivity and synchronization between the embryo and the endometrium [8–10].

Numerous strategies and interventions have been used as adjuvant treatments to enhance endometrial receptivity, including estradiol hormonal supplementations, angiogenesis regulators, antioxidants, immunomodulators, etc. [11, 12]. Meanwhile, intrauterine infusion treatments get the most attention for their safety, simplicity, and operation friendliness. Intra-uterine infusion of human chorionic gonadotropin (HCG), autologous peripheral blood mononuclear cells (PBMCs), granulocyte colony-stimulating factor (G-CSF) or Platelet-rich plasma (PRP) were reported to be beneficial to embryo implantation and pregnant outcomes, considered as potential therapies for RIF [13–16]. Nevertheless, their efficiencies were controversial, with some advocating improvement, and others showing no benefit [17–19]. Furthermore, most of the previous studies compared the effects of two treatments, and few studies compared multiple treatments at the same time. Besides, insufficient attention has been attached to new but less-applied infusion strategies, such as combination therapy. Consequently, there lack of consensus on which intrauterine treatment is the most effective, and unable to give good guidance for the clinical treatment.

Network meta-analysis (NMA) is probably an effective method to explore the most effective uterine-infusion treatment, for it can compare three or more treatments simultaneously, integrating the results of direct and indirect comparisons, summarizing the possibility values of the efficacy of each treatment, and reporting the best option. Network meta-analyses have been used to explore the best IUI protocol for unexplained infertility [20], compare the efficiency of diverse luteal phase support [21], and evaluate the role of different adjuvant treatment strategies on the probability of pregnancy achievement in poor responders undergoing IVF [22], etc.

It remains a challenge for clinicians to treat repeated IVF failures characterized by no anatomical pathologies, good response to treatment, and good embryo quality but no occurrence of pregnancy. Therefore, the study aimed to screen research that compared the effects of different intrauterine infusion treatments in women with RIF. Furthermore, a network meta-analysis is performed to comprehensively evaluate the improvement of pregnant outcomes. The research aims to rank the efficacy of each uterine infusion strategy in different pregnancy outcomes to guide clinical decision-making for women with repeated implantation failure.

## Methods

### Literature search and study selection

We reported and conducted a systematic review and network meta-analysis based on the guidelines of the preferred reporting items for systematic reviews and meta-analyses (PRISMA). The PubMed, Cochrane Library, CNKI, and Web of Science databases were systematically searched from July 2013 to July 2023. MeSH search combined with random word search was used in our study. The search items included “Chorionic Gonadotropin”, “Granulocyte Colony-Stimulating Factor”, “G-CSF”, “Monocytes”, “PBMC”, “Platelet-Rich Plasma”, “PRP”, “Lymphocytes”, “Uterine perfusion”, “RIF”, “ Repeated implantation failure”, “Recurrent implantation failure”. Meanwhile, we also checked references listed in the included studies and all related reviews and guidelines to supplement any previously ignored literature. Our study was registered in PROSPERO and the ID was CRD42023467188.

Prospective trials (in Chinese or English) were included if they met the following criteria: 1. Objectives of the study were RIF patients. 2. At least one of the uterine perfusion treatments was included. 3. Reported at least one of the pregnant outcomes: chemical pregnancy, clinical pregnancy, implantation, miscarriage, and live birth. Two independent investigators (Q.X. and XZ.Q.) identified and evaluated all eligible studies. Registration of all subjects or ethical approval is not applicable because the data used by this meta-analysis were from published studies.

### Quality assessment of risk of bias

The methodology and categories described in the Cochrane Collaboration Handbook were used to assess the risk of bias in RCT studies. The risks of bias graphs were constructed with Review Manager 5.3 software.

The risk of bias in non-random controlled court and prospective observe study was assessed by the methodological index for non-randomized studies (MINORS) and Newcastle–Ottawa Scale (NOS) respectively.

Two independent investigators (QX and XZY) assessed the risk of bias. The other investigators YLL and XZQ would attend if any discrepancy existed.

### Data extraction

Extracted data included: 1. Characteristics of study (authors and publication year); 2. Patient characteristics (RIF definition, inclusion and exclusion criteria); 3. Trial design details (sample size, uterus perfusion methods, embryo transfer method, embryo grades, pregnant outcome). Two independent investigators (Q.X. and XZ. Q.) extracted the relevant data. If there existed any discrepancies, reviewer XZ. Y. would attend to and resolve them by consensus of the reviewers.

### Data synthesis and statistical analysis

Traditional direct pairwise comparisons were performed by Review Manager 5.3 software if direct data were available. The pregnant outcomes would be analyzed if the amount of relevant research was not less than three. We synthesized data and calculated summary odds ratios (ORs) and 95% CIs. If heterogeneity index I^2^ ≥ 50%, and *P* < 0.05, a random effect model was applied, otherwise, a fixed effect model was used. Funnel plots were used to detect publication bias.

The network meta-analysis was conducted by the STATA software package (version 15.0, StataCorp, College Station, TX) and the GeMTC software package. STATA software package portrayed a network of eligible comparisons. All trials with recurrent implantation failure were included in the network meta-analysis. Before conducting the network meta-analysis, consistency and convergence were assessed. We verified inconsistencies by the node splitting method, which separated evidence for a particular comparison into direct and indirect evidence, and indicated a significant inconsistency. An inconsistency model was used if inconsistency *P* < 0.05 was identified, otherwise, the relative effects of the interventions were analyzed using a consistency model. We used the Brooks-Gelman-Rulin method to assess the Convergence: the potential scale reduction factors (PSRF) were close to 1 for all of the chains, and the results were considered to be well-converged. We summarized the possibility values of the efficacy of each treatment and reported the best option and surface under the cumulative ranking curve (SUCRA). The value of SUCRA ranges from 0 to 100%, and the higher the score is, the more likely it is that the treatment will be the best option.

Besides, publication bias was analyzed by the funnel plot, and sensitivity analysis was conducted according to the origin of the publication.

## Results

### Selection and characteristics of the included studies

**Fig. 1:**
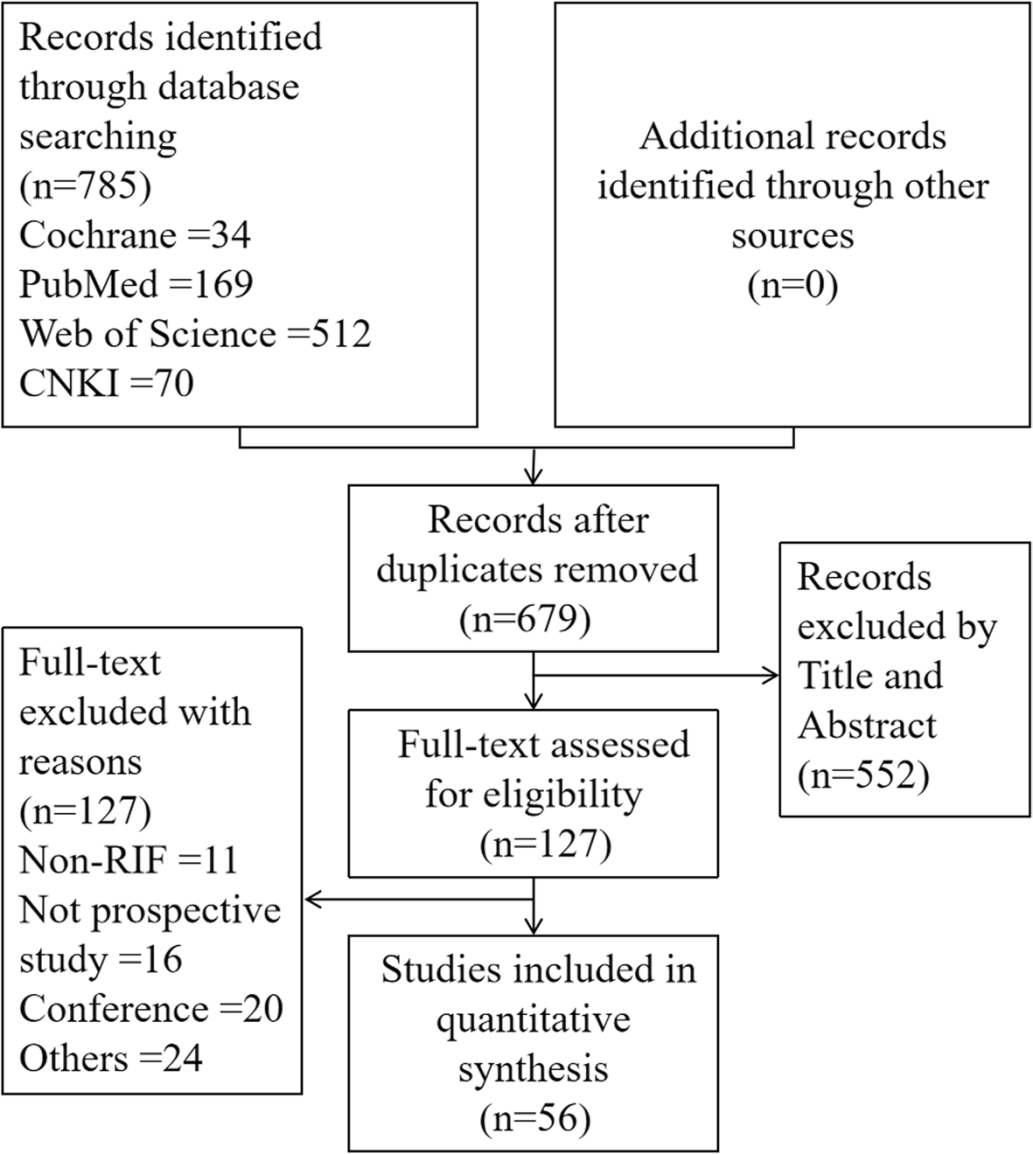
Flow diagram for identification and selection of studies

After electronic searches, 679 potential citations were identified. The selection process is shown in Fig. 1. Subsequently, a total of 56 studies were included: 40 were randomized controlled trials (RCTs) [19, 23–61], 13 were non-RCTs [48, 62–73], and 3 were prospective cohort studies [74–76]. Meanwhile, 31 were published in English and 25 in Chinese. The control group was blank without any uterine infusion. Comparative efficacies of 11 therapies were conducted: Placebo, Human Chorionic Gonadotropin (HCG), Granulocyte Colony-Stimulating Factor (G-CSF), Platelet-rich plasma (PRP), Peripheral Blood Mononuclear Cell (PBMC), Growth hormone (GH), Dexamethasone (DEX), Embryo culture supernatant (ESC), PRP combined with G-CSF (PRP + G-CSF), RPR combined with subcutaneous injection of G-CSF (RPR + G-CSFsc), G-CSF combined with subcutaneous injection of AXaIU (G-CSF + AXaIUsc). The main characteristics of the studies included are presented in Supplemental Table S1. The number of subjects ranges from 37 to 393. Among a total of 56 studies, 12, 14, 15, and 19 studies involved HCG, G-CSF, PRP, and PBMC, respectively. Only one study involved GH, DEX, ESC, PRP + G-CSFsc, PRP + G-CSF, or G-CSF + AXaIUsc.

### Risk of bias assessment results

The risk of bias of 40 RCTs was assessed by the Cochrane Collaboration Handbook, as shown in Supplemental Figure S1. 31(76%) researches were with low risk in random sequence generation. 17(43%) researches showed a high risk of bias with "Blind of participants and personnel", 15(38%) researches with low risk, and 8(20%) researches with unclear. Allocation concealment was not referred to in 28(70%) researches and 11 (28%) researches with low risk in allocation concealment. Other terms were shown with low risk.

MINORS questionnaire was performed to evaluate the risk of bias in 13 non-randomized controlled trials, as shown in Supplemental Table S2a. All studies did not estimate the sample size, which might increase the risk of bias and decrease the incredibility.

We assessed the risk of bias in 3 prospective cohort studies by NOS questionnaire (Supplemental Table S2b). All these researches seemed of high quality.

### Meta-analysis results

#### Primary outcome measure: clinical pregnancy rate

**Fig. 2:**
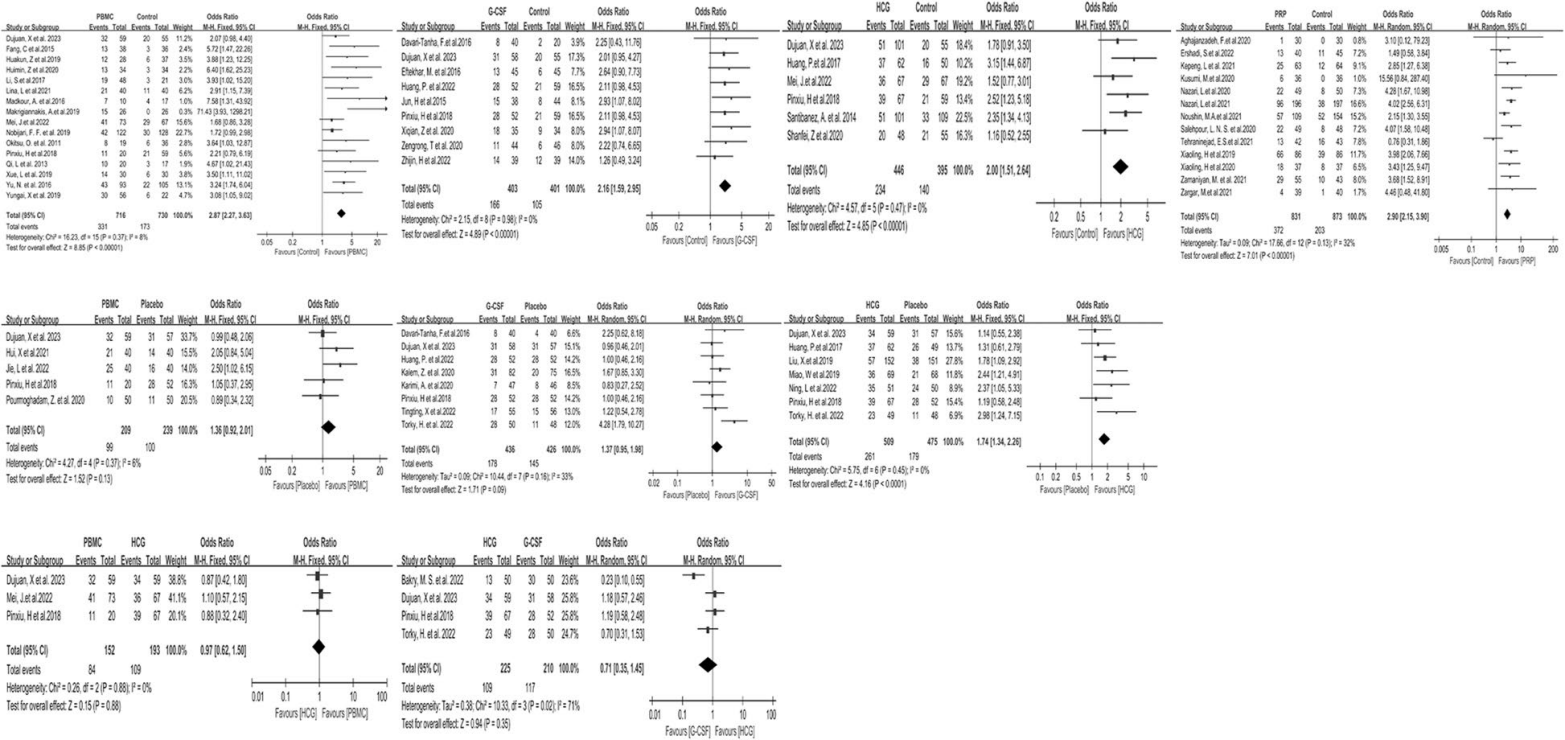
Forest plot of the clinical pregnancy in direct pair-wise meta-analysis

The outcome of the direct pairwise meta-analysis is shown in Fig. 2. Compared with the control group, PBMC, G-CSF, HCG, and PRP could significantly increase the clinical pregnancy rate (OR 2.87, 95% CI 2.27 to 3.63; 2.16, 1.59 to 2.95; 2.00, 1.51 to 2.64; 2.90, 2.15 to 3.90; respectively). Only HCG (OR 1.74, 95% CI 1.34 to 2.26), but not the PBMC or the G-CSF group (OR 1.36, 95% CI 0.92 to 2.01; 1.37, 0.95 to 1.98, respectively) with a higher clinical pregnancy rate than the placebo group with statistical significance. The clinical pregnancy rate between the HCG and the PBMC group, G-CSF and HCG group were comparable (OR 0.97, 95% CI 0.62 to 1.50; 0.71, 0.35 to 1.45, respectively).

**Fig. 3:**
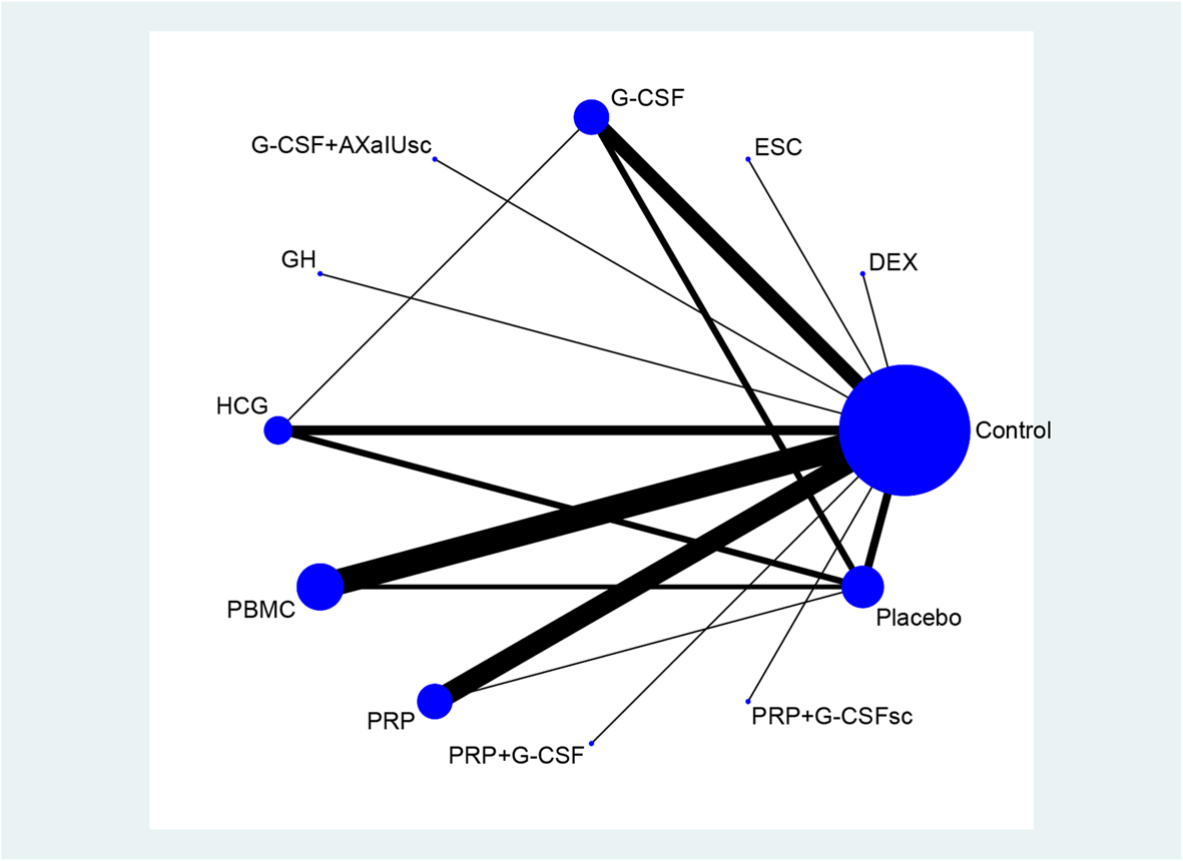
Network plots of eligible comparisons for primary outcomes: clinical pregnancy rate

The results of the network meta-analysis are shown in Table 1 and Fig. 3. When compared to the control group, except the ECS and PRP + GCSFsc group, almost all uterine infusions significantly improve the clinical pregnancy including DEX (OR 2.76, 95% CI 1.16 to 5.81)), G-CSF (OR 2.58, 95% CI 1.90 to 3.43)), GCSF + AXaIUsc (OR 3.34, 95% CI 1.38 to 8.19)), GH (OR 2.80, 95% CI 1.14 to 6.60)), HCG (OR 2.27, 95% CI 1.76 to 3.01)), PBMC (OR 3.03, 95% CI 2.42 to 4.07)), PRP (OR 3.07, 95% CI 2.30 to 3.88)), PRP + GCSF (OR 2.90, 95% CI 1.19 to 10.06)), and placebo group(OR 1.59, 95% CI 1.18 to 2.06)). Meanwhile, OR for clinical pregnancy rates in placebo was significantly inferior to PRP (OR 0.49, 95% CI 0.37 to 0.76), PBMC (OR 0.52, 95% CI 0.37 to 0.67), HCG (OR 0.69, 95% CI 0.51 to 0.89), G-CSF (OR 0.62, 95% CI 0.46 to 0.85) respectively. Furthermore, OR for clinical pregnancy rates comparing PBMC to ECS and HCG was 2.22 (95% CI 1.14 to 5.23) and 1.36 (95% CI 1.00 to 1.89). OR for clinical pregnancy rates comparing PRP to ECS was 2.40(95% CI 1.07 to 4.94).

**Table 1:** Network meta-analysis for clinical pregnancy comparing diverse uterine infusion strategies

Groups/pregnant outcomes	DEX	ECS	G-CSF	G-CSF + AXaIUsc	GH	HCG	PBMC	PRP	PRP + G-CSF	PRP + G-CSFsc	Placebo
Control	2.76 (1.16, 5.81)	1.36 (0.64, 2.64)	2.58 (1.90, 3.43)	3.34 (1.38, 8.19)	2.80 (1.14, 6.60)	2.27 (1.76, 3.01)	3.03 (2.42, 4.07)	3.07 (2.30, 3.88)	2.90 (1.19, 10.06)	1.51 (0.41, 4.59)	1.59 (1.18, 2.06)
DEX		0.46 (0.17, 1.47)	0.88 (0.41, 2.35)	1.16 (0.39, 4.34)	0.95 (0.32, 3.44)	0.83 (0.37, 2.07)	1.11 (0.52, 2.80)	1.09 (0.49, 2.67)	1.09 (0.31, 5.09)	0.49 (0.13, 2.06)	0.56 (0.25, 1.43)
ECS			1.89 (0.90, 4.28)	2.46 (0.86, 7.94)	2.17 (0.66, 6.45)	1.67 (0.83, 3.81)	2.22 (1.14, 5.23)	2.40 (1.07, 4.94)	2.22 (0.74, 9.51)	1.08 (0.25, 4.05)	1.17 (0.56, 2.55)
G-CSF				1.31 (0.54, 3.18)	1.11 (0.46, 2.55)	0.89 (0.66, 1.29)	1.19 (0.89, 1.75)	1.23 (0.79, 1.71)	1.16 (0.45, 3.97)	0.60 (0.15, 1.99)	0.62 (0.46, 0.85)
G-CSF + AXaIUsc					0.87 (0.25, 2.65)	0.64 (0.28, 1.72)	0.89 (0.38, 2.33)	0.94 (0.35, 2.24)	0.87 (0.25, 3.84)	0.45 (0.09, 1.98)	0.48 (0.19, 1.15)
GH						0.79 (0.35, 2.09)	1.08 (0.47, 2.85)	1.13 (0.43, 2.68)	1.05 (0.33, 4.68)	0.51 (0.10, 2.40)	0.56 (0.23, 1.38)
HCG							1.36 (1.00, 1.89)	1.35 (0.88, 1.98)	1.26 (0.51, 4.43)	0.66 (0.17, 2.04)	0.69 (0.51, 0.89)
PBMC								1.00 (0.67, 1.36)	0.95 (0.37, 3.27)	0.50 (0.12, 1.54)	0.52 (0.37, 0.67)
PRP									0.96 (0.36, 3.47)	0.44 (0.13, 1.54)	0.49 (0.37, 0.76)
PRP + G-CSF										0.50 (0.07, 2.02)	0.53 (0.15, 1.37)
PRP + G-CSFsc											1.06 (0.32, 3.93)

When ranked from the best therapy, it was shown that uterine infusion with PRP + GCSF was the most effective (SUCRA 26%), followed by GCSF + AXaIUsc (SUCRA 20%), PBMC (SUCRA 22%), and PRP (SUCRA 20%), as shown in Table 2.

**Table 2:** A comprehensive sorting table for all the outcomes

Rank	Clinical pregnancy	Live birth	Implantation	Chemical pregnancy	Miscarriage
1	PRP + G-CSF (0.26)	GH (0.13)	GH (0.38)	PRP + G-CSF (0.47)	DEX (0.91)
2	G-CSF + AXaIUsc (0.20)	HCG (0.21)	PBMC (0.21)	PRP (0.18)	PRP + G-CSFsc (0.63)
3	PBMC (0.22)	G-CSF (0.22)	PRP (0.15)	G-CSF (0.22)	GH (0.27)
4	PRP (0.20)	Placebo (0.23)	G-CSF (0.22)	HCG (0.18)	Placebo (0.25)
5	G-CSF (0.19)	PBMC (0.23)	G-CSF + AXaIUsc (0.10)	Placebo (0.19)	Control (0.32)

#### Secondary outcome measures: live birth

The direct pair-wise meta-analysis (Supplemental Figure S2) showed that G-CSF, PBMC, and PRP significantly increased the live birth compared with the control group (OR 2.90, 95% CI 1.68 to 5.00; 2.23, 1.25 to 4.00; 4.27, 1.25 to 14.66; respectively). However, no significant difference was found between the placebo and the G-CSF group (OR 1.28, 95% CI 0.65 to 2.51).

The outcome of the network meta-analysis showed that, compared to the control group (Supplemental Table S3 and Figure S3), the PRP group (OR 4.85, 95% CI 1.76 to 14.56) significantly improved the live birth rate. Others including G-CSF, GH, HCG, PBMC, PRP + GCSFsc, and Placebo group didn't significantly increase the live birth rate. There existed no significant difference in live births among all uterine treatments.

When ranked from the best therapy, it was shown that uterine infusion with GH was the most effective (SUCRA 13%), followed by HCG (SUCRA 21%), GCSF (SUCRA 22%), and Placebo (SUCRA 23%) (Table 2).

#### Secondary outcome measures: embryo implantation

The direct pair-wise meta-analysis (Supplemental Figure S4) showed that, compared with the control group, PBMC, HCG, G-CSF, and PRP significantly increased the implantation (OR 2.43, 95% CI 1.89 to 3.13; 1.47, 1.05 to 2.05; 2.20, 1.62 to 2.99; 2.64, 1.72 to 4.07; respectively). The effect persisted in PBMC and HCG even when compared with the placebo group (OR 1.52, 95% CI 1.03 to 12.22; 1.65, 1.29 to 2.12).

In the network meta-analysis, when compared to the control group (Supplemental Table S4 and Figure S3), G-CSF (OR 2.62, 95% CI 1.89 to 3.74), GCSFAIU (OR 2.81, 95% CI 1.20 to 6.46), GH (OR 3.57, 95% CI 1.53 to 8.25), HCG (OR 1.94, 95% CI 1.33 to 2.79), PBMC (OR 3.03, 95% CI 2.25 to 4.32), PRP (OR 2.77, 95% CI 1.55 to 4.83) significantly improved the clinical pregnancy. Meanwhile, OR for implantation rates comparing PBMC to HCG was 1.57 (95% CI 1.05 to 2.42).

When ranked from the best therapy, it was shown that uterine infusion with GH was the most effective (SUCRA 38%), followed by PBMC (SUCRA 21%), PRP (SUCRA 15%) and G-CSF (SUCRA 22%) (Table 2).

#### Secondary outcome measures: chemical pregnancy

The direct pair-wise meta-analysis (Supplemental Figure S5) showed that the chemical pregnancy in the PRP group was significantly higher than the control group (OR 2.41, 95% CI 1.92 to 3.03). However, no significant difference was found between the placebo and the G-CSF group in terms of chemical pregnancy.

In the network meta-analysis, when compared to the control group (Supplemental Table S5 and Figure S3), the PRP group (OR 1.90, 95% CI 1.00 to 3.54) significantly improved the chemical pregnancy. Others including G-CSF, GH, HCG, PBMC, PRP + GCSF, and Placebo group didn’t significantly increase the implantation rate. No significant difference in chemical pregnancy among all uterine treatments.

When ranked from the best therapy, it was shown that uterine infusion with PRP + GCSF was the most effective (SUCRA 47%), followed by PRP (SUCRA 18%), GCSF (SUCRA 22%) and HCG (SUCRA 18%) (Table 2).

#### Secondary outcome measures: miscarriage

In the direct pair-wise meta-analysis (Supplemental Figure S6), G-CSF (OR 0.28, 95% CI 0.13 to 0.59), but not the PBMC and HCG could significantly decrease the miscarriage rate compared with the control group. When compared with the placebo group, PBMC and G-CSF (OR 0.24, 95% CI 0.11 to 0.56; 0.24, 0.12 to 0.49), but not the HCG significantly decreased the miscarriage rate. Besides, there was no difference between the PBMC and HCG, HCG and G-CSF group in terms of miscarriage.

In the network meta-analysis, when compared to the control group (Supplemental Table S6 and Figure S3), the DEX group significantly increased the miscarriage rate, but the GCSF and PBMC group significantly decreased the miscarriage rate (OR 0.29, 95% CI 0.14 to 0.64; 0.38, 0.19 to 0.83). The miscarriage in the Placebo group was significantly higher than in the PBMC, HCG, and GCSF group (OR 2.82, 95% CI 1.40 to 6.10; 1.79, 1.03 to 3.50; 3.79, 1.92 to 8.25; respectively). Meanwhile, OR for miscarriage rates in PRP was significantly higher than G-CSF (OR 3.36, 95% CI 1.03 to 8.73).

When ranked, it was shown that uterine infusion with DEX had the highest probability of miscarriage (SUCRA 91%), followed by PRP + GCSFsc (SUCRA 63%), GH (SUCRA 27%), and Placebo (SUCRA 25%) (Table 2).

#### Heterogeneity and publication bias

During data merging, most studies showed low heterogeneity, except for the outcomes of clinical pregnancy and implantation between HCG and G-CSF, live birth between the PRP and control, and implantation and chemical pregnancy between G-CSF and placebo. The inadequate quantity (≤ 5) of research might be one of the reasons for the heterogeneity. On the other hand, the overall publication bias appears low as analyzed by the funnel plot, as shown in Supplemental Figure S7.

#### Sensitivity analysis

We did sensitivity analysis according to the origin of the publication: English and Chinese.

In the subgroup analysis by English researches, compared with the control group (Supplemental Figure S8), HCG (OR 2.20, 95% CI 1.51 to 3.21), G-CSF (OR 2.28, 95% CI 1.27 to 4.07), PBMC (OR 2.66, 95% CI 1.95 to 3.63), and PRP (OR 2.83, 95% CI 2.20 to 3.64) infusion could significantly increase the clinical pregnancy. HCG group and G-CSF group had a higher clinical pregnancy than the placebo group as well (OR 1.82, 95% CI 1.25 to 2.64; 1.68, 1.15 to 2.48). In the network meta-analysis (Supplemental Table S7), G-CSF, HCG, PBMC, PRP, PRP + G-CSFsc, and placebo could significantly increase the clinical pregnancy than the control group. The clinical pregnancy rate in the placebo group was significantly lower than in the G-CSF and PBMC groups.

In the subgroup analysis by Chinese researches, compared with the control group (Supplemental Figure S9), HCG (OR 1.78, 95% CI 1.17 to 2.69), G-CSF (OR 2.12, 95% CI 1.47 to 3.06), PBMC (OR 3.17, 95% CI 2.22 to 4.52), and PRP (OR 3.47, 95% CI 2.20 to 5.47) infusion could significantly increase the clinical pregnancy. HCG (OR 1.67, 95% CI 1.15 to 2.40) but not the G-CSF and the PBMC (OR 1.05, 95% CI 0.67 to 1.63; 1.48, 0.96 to 2.27) was with a higher clinical pregnancy than the placebo group. In the network meta-analysis (Supplemental Table S8), DEX, G-CSF, G-CSF + AIU, GH, HCG PBMC, PRP, PRP + G-CSF and Placebo but not the ESC could significantly increase the clinical pregnancy than the control group. The clinical pregnancy in the PBMC and PRP groups was significantly higher than in the ESC group. The placebo group had a lower clinical pregnancy than the HCG, PBMC, and PRP groups.

## Discussion

In the traditional pairwise meta-analysis, HCG, PBMC, G-CSF, and PRP treatments improved pregnancy outcomes no matter compared with the control group or the placebo group. Meanwhile, there existed no significant difference between PBMC and HCG in terms of clinical pregnancy and miscarriage, and between HCG and G-CSF in terms of implantation, clinical pregnancy, and miscarriage. In the network meta-analysis, no one treatment showed absolute dominance among pregnancy outcomes but the PRP improved the clinical pregnancy and live birth most. In terms of clinical pregnancy, PBMC was superior to HCG and ECS, and PRP was superior to ECS. PBMC was superior to HCG at implantation. DEX treatment showed high risk of miscarriage, and the risk of miscarriage of PRP is higher than G-CSF treatment. No significant difference was found among intrauterine infusion treatments in chemical pregnancy and live birth. When it comes to the outcomes rank, intrauterine infusion with PRP + GCSF ranked highest in clinical pregnancy, GH ranked highest in embryo implantation, PRP + GCSF ranked highest in chemical pregnancy, DEX ranked highest in miscarriage, GH ranked highest in live birth. The probability of rank for pregnant outcomes is rather low except for the miscarriage, it should be interpreted with caution. In subgroup analysis by the origin of the publication, the outcome was similar.

Several uterine infusion strategies have been proposed for improving pregnancy outcomes for RIF patients, especially HCG, G-CSF, PBMC, and PRP. These treatments were reported to mediate the balance of immune cells and the abnormal cytokine secretion, anti-apoptosis, promote angiogenesis, and so on [28, 77–80].

In our study, the number of research about HCG, PBMC, G-CSF, and PRP were the most, indicating they were possibly effective treatments from researchers' perspectives. The pair-wise comparison showed that pregnant outcomes were significantly improved by HCG, PBMC, G-CSF, and PRP treatments compared with the control group or the placebo group, which were consistent with other studies.

The study of Xie, H. et al. included six trials, of 1432 women to explore the efficiency of intrauterine perfusion of hCG [14]. They found HCG infusion could significantly improve the clinical pregnancy rate and live birth rate compared with no perfusion of HCG. Implantation, pregnancy, and live birth rates were significantly increased in the group of PBMCs treatment. But Pourmoghadam, Z. et al. also found that the miscarriage rate was significantly decreased in the PBMC-treated group than the non-treated group, which did not reach significance in our study [16]. A meta-analysis consisting of 20 RCTs found that G-CSF increased the biochemical pregnancy rate, embryo implantation rate, and clinical pregnancy rate [81]. Another meta-analysis found G-CSF administration may improve clinical pregnancy rate, but it concluded that they were uncertain whether the administration of G-CSF improves ongoing pregnancy or overall clinical pregnancy rates or reduces miscarriage rate compared to no treatment or placebo for the included studies had unclear allocation concealment or were at high risk of performance bias [18]. As regards the efficiency of PRP, the rates of clinical pregnancy, chemical pregnancy, live births, and implantation were significantly higher than in the control group [82–84]. A network analysis published recently compared the effect of PRP, G-CSF, PBMC, and HCG intrauterine infusion on the pregnant outcomes of RIF patients [85]. Its outcomes confirmed that all four intrauterine infusion drugs can improve pregnancy outcomes in RIF patients to varying degrees, with PRP being the most effective, which was similar to our study.

GH, DEX, and ECS intrauterine infusions were also included in our study, but the amount of research was few, and no direct comparisons with other infusion treatments exist. Zihua Wang et al. indicated that DEX could significantly improve embryo implantation and clinical pregnancy, and have no significant effect on miscarriage [23]. However, outcomes of the network meta-analysis combining all the direct and indirect comparisons showed that DEX could significantly increase the possibility of miscarriage, so the use of DEX needs more consideration. Embryos are reported to secrete certain factors during development. These factors might affect the process of implantation and predict the successful embryo implantation [86–88]. The implantation rate may be improved by the performance of intrauterine perfusion of embryo culture supernatant before embryo transfer [65]. There was only one study about the effect of GH as well. GH treatment was shown to significantly improve the implantation compared with the control group and the effect seems to be superior to the G-CSF treatment group [60]. Outcomes of network meta-analysis showed that implantation and clinical pregnancy were significantly improved by GH infusion. Meanwhile, GH infusion performed the best in the efficient ranking of the embryo implantation rate and live birth rate. However, the probabilities of the ranking were universally low, requiring further verifications.

In addition, the combination treatments did not show obvious advantages. No one combination of treatments significantly improved the pregnant outcomes with comparisons to the HCG, PBMC, G-CSF, or PRP treatment. In terms of the effectiveness ranking, the biochemical pregnancy rate and clinical pregnancy rate in the G-CSF + PRP were the highest. However, the ranking outcomes were with low probabilities, which should be interpreted with caution. More high-quality researches are required to further verify the efficiency of combination treatments.

There are some strengths in our study. First, we showed the overview of the various uterine infusion treatments, which also included new but less-applied infusion strategies, such as combination therapy. Meanwhile, main pregnancy outcomes including implantation, chemical pregnancy, clinical pregnancy, miscarriage, and live birth were explored. Second, a network meta-analysis was performed, which can apply indirect comparison to draw comparison outcomes and provide the opportunity to rank the different treatment strategies in order of effectiveness, facilitating clinical decision-making. Third, the number of researches included was relatively large for we included both English and Chinese articles, which might contribute to accurate results. Furthermore, subgroup analysis by the publication origin was conducted.

Some limitations must be noted. First, there was no universe definition of RIF, 25 researches (45%) in our study considered RIF patients to be women under the age of 40 years who have failed to achieve a clinical pregnancy after the transfer of at least four good-quality embryos in a minimum of three IVF fresh or frozen cycles. 19 researches (34%) defined RIF as patients failed transfer of good-quality embryos at least once, more than twice, or twice to more than three times. 12 researches (21%) did not clearly describe the definition. Besides, we included RCT, non-RCT trials, and prospective cohort trials, which might lead to some heterogeneity. Second, variations existed in terms of infusion time point and times of infusion, ET cycle, endometrium preparation, etc. Meanwhile, no subgroup analysis was further conducted to decrease the bias of confounding factors such as age, which is an essential factor in deciding the pregnant outcomes of patients with recurrent implantation failure. Third, meta-analyses for safety were not able to be performed due to inadequate research. The development and health of children delivered under the related treatment should also be focused.

## Conclusion

Intrauterine infusion of human chorionic gonadotropin, peripheral blood mononuclear cells, granulocyte-colony-stimulating factor, and platelet-rich plasma seems a promising strategy for improving the pregnant outcomes for infertile patients with recurrent implantation failure. Among these treatments, PRP may be the best. More researches are required to explore the effect of drug combinations and less commonly used drugs as well.

### Supplementary Information

**Additional file 1: Figure S1.** Risk of bias assessment. a. Risk of bias summary; b. Risk of bias graph. **Figure S2.** Forest plot of the live birth in direct pair-wise meta-analysis. **Figure S3.** Network plots of eligible comparisons for secondary outcomes: clinical pregnancy rate. a. Live birth; b. Embryo implantation; c. Chemical pregnancy; d. Miscarriage. **Figure S4.** Forest plot of the embryo implantation in direct pair-wise meta-analysis. **Figure S5.** Forest plot of the chemical pregnancy in direct pair-wise meta-analysis. **Figure S6.** Forest plot of the miscarriage in direct pair-wise meta-analysis. **Figure S7.** Funnel plot of the pregnancy outcomes. **Figure S8.** Subgroup analysis of forest plot of the clinical pregnancy in the direct pair-wise meta-analysis by English researches. **Figure S9.** Subgroup analysis of forest plot of the clinical pregnancy in the direct pair-wise meta-analysis by Chinese researches. **Supplemental Table S1.** Characteristics of studies included in meta-analyses. **Supplemental Table S2.** Risk of bias assessment of the other prospective studies. **Supplemental Table S3.** Network meta-analysis for live birth comparing diverse uterine infusion strategies. **Supplemental Table S4.** Network meta-analysis for implantation comparing diverse uterine infusion strategies. **Supplemental Table S5.** Network meta-analysis for chemical pregnancy comparing diverse uterine infusion strategies. **Supplemental Table S6.** Network meta-analysis for miscarriage comparing diverse uterine infusion strategies. **Supplemental Table S7.** Subgroup analysis of network meta-analysis for clinical pregnancy by English researches. **Supplemental Table S8.** Subgroup analysis of network meta-analysis for clinical pregnancy by Chinese researches.

## Data Availability

No datasets were generated or analysed during the current study.
