# Supplementary material for: Uterine infusion strategies for infertile patients with recurrent implantation failure: a systematic review and network meta-analysis
Source: Reprod Biol Endocrinol. 2024 Apr 16;22:44. doi: 10.1186/s12958-024-01221-x (PMC11020641; doi:10.1186/s12958-024-01221-x)
Supplement: Supplementary file 1 — Additional file 1: Figure S1. Risk of bias assessment. a. Risk of bias summary; b. Risk of bias graph. Figure S2. Forest plot of the live birth in direct pair-wise meta-analysis. Figure S3. Network plots of eligible comparisons for secondary outcomes: clinical pregnancy rate. a. Live birth; b. Embryo implantation; c. Chemical pregnancy; d. Miscarriage. Figure S4. Forest plot of the embryo implantation in direct pair-wise meta-analysis. Figure S5. Forest plot of the chemical pregnancy in direct pair-wise meta-analysis. Figure S6. Forest plot of the miscarriage in direct pair-wise meta-analysis. Figure S7. Funnel plot of the pregnancy outcomes. Figure S8. Subgroup analysis of forest plot of the clinical pregnancy in the direct pair-wise meta-analysis by English researches. Figure S9. Subgroup analysis of forest plot of the clinical pregnancy in the direct pair-wise meta-analysis by Chinese researches. Supplemental Table S1. Characteristics of studies included in meta-analyses. Supplemental Table S2. Risk of bias assessment of the other prospective studies. Supplemental Table S3. Network meta-analysis for live birth comparing diverse uterine infusion strategies. Supplemental Table S4. Network meta-analysis for implantation comparing diverse uterine infusion strategies. Supplemental Table S5. Network meta-analysis for chemical pregnancy comparing diverse uterine infusion strategies. Supplemental Table S6. Network meta-analysis for miscarriage comparing diverse uterine infusion strategies. Supplemental Table S7. Subgroup analysis of network meta-analysis for clinical pregnancy by English researches. Supplemental Table S8. Subgroup analysis of network meta-analysis for clinical pregnancy by Chinese researches. [file 12958_2024_1221_MOESM1_ESM.zip › Table S1.docx]

**Supplemental Table S1** Characteristics of studies included in meta-analyses.

| **Groups** | **Study** | **Research type** | **Research time** | **inclusion criteria** | **exclusion criteria** | **ART** | | **Infusion treatment** | | |
| --- | --- | --- | --- | --- | --- | --- | --- | --- | --- | --- |
|  |  |  |  |  |  | **Embryo** | **fET/FET** | **time** | **dose** | **frequency** |
| DEX  Control | Zihua W.et al.(2021) | RCT | From June 2018 to October 2020 | RIF | uterine malformation; History of uterine adhesions and endometritis; Chromosomal abnormalities; endometriosis; serious endocrine abnormalities;  abnormal coagulation function. | High-quality embryos and blastocysts | FET | On the day of progesterone administration | 5 mg  (1 mL) | 1 |
| Hcg  placebo | Liu, X.et al.(2019) | Prospective cohort study | January 1st 2016 to December 31st 2016 | a history of RIF, age ≤ 45 years, 19≤BMI≤30 kg/m2, basal FSH<10 IU/L, normal uterine cavity, normal maternal and paternal karyotypes | severe uterine malformation, severe uterine adhesions, chromosomal abnormality, antiphospholipid syndrome, hydrosalpinx, any contraindication to pregnancy, thyroid or adrenal dysfunction, neoplasia, severe impairment of renal or hepatic function, and use of medications that might interfere with study evaluations | Day 3, day 5 or day 6 embryos | FET | 3 days before embryo transfer | 50 µl (500 IU) | 1 |
| Hcg  control | Santibanez, A.et al.(2014) | RCT | August 2011 through November 2012 | RIF | azoospermia | cleavage embryo | fET and FET | On the ET day before embryo transfer | 0.2 mL (500 IU) | 1 |
| Hcg  controlplacebo | Huang, P.et al.(2017) | RCT | January 2015 and December 2015 | With 2 instances of failed implantation of goodquality embryos, aged≤38 years, 18 ≤BMI≤ 24, normal endometrial thickness (8–16 mm), frozen preservation of two or more embryos, with at least one goodquality embryo. | With diseases, such as endometrial polyps, intrauterine adhesion, or uterine submucosal myomas, that might cause endometrial abnormalities; adenomyosis; patients who had hydropic fallopian tubes, PCOS, or endometriosis of stage III or higher. | two cleavage embryos, at least one of which was a goodquality embryo | FET | 3 days before ET | 1mL  (1000 IU) | 1 |
| Hcg  placebo | Ning Liu et al.(2022) | Non-RCT | May 2019 to May 2020 | previous transplantation cycles is ≥3, and there are ≥4 good quality embryos, but clinical pregnancy is still not obtained; Both spouses have normal chromosomes; Frozen embryos at cleavage stage were transplanted, and there was at least one high quality embryo; Age < 40 years old; | Infertility caused by tubal cyst, uterine fibroid and other factors; Positive antiphospholipid antibody; the uterus has organic lesions; hydrosalpinx; Uncontrolled endocrine and metabolic diseases. | day 3 of cleavage stage | FET | 15min before FET | 1mL  (1 000U) | 1 |
| Hcg  control | Shanfe Zhao et al. (2020) | RCT | NA | All the infertile patients were transplanted more than 3 times or transplanted 46 embryos with high blastomere score or 3 or more blastocysts with high blastomere score, age≤38,18≤BMI≤24kg/m2, two or more cleavage embryos can be transferred | There are other factors that affect the implantation of embryos, such as adenomyosis, endometriosis, uterine malformation, endometrial abnormalities, hydrosalpinx, and uterine adhesions. | cleavage embryo | FET | one day before FET | 0.5 ml  (500U) | 1 |
| Hcg  placebo | Miao Wang et al. (2019) | RCT | April 2014 to November 2017 | Embryo transfer ≥3 cycles; Cumulative number of transplanted embryos ≥4; at least 1 highquality embryo per transfer; no clinical pregnancy was obtained, age <40; regular menstruation. | 1)Uterine abnormalities: intermuscular myoma (≥3 cm), submucosal myoma, adenomyoma, endometrial polyps, endometrial dysplasia, uterine malformations, uterine adhesions and abnormal uterine morphology Hydrosalpinx, endometriosis; Chromosomal abnormalities in both or one of the couples; The transferred embryos are blastocyst stage embryos, or embryos that have undergone preimplantation genetic tests. | cleavage embryo | FET | One day of Frozen/thawed embryo transfer | 40 μL | 1 |
| PBMC  control | Li, S.et al.(2017) | NonRCT | July 2013 and March 2015 | All patients had experienced at least one failures of IVFembryo transfer therapy; Clinical indications for IVF/ICSI included tubal and pelvic factors, mild and moderate endometriosis, ovulation failure, sperm abnormalities and mixed factors. | Patients who had poor ovarian reserve (FSH >15 mIU/ml), thin endometrium (endometrium was <7 mm on day of ET), chromosomal abnormalities, presence of antiphospholipid antibodies, or mutations involving parts of the coagulation system, such as deficiencies of factor XII, protein C, and protein S | cleavage embryo or blastocyst | FET | One day before FET | ((1–2 × 10^7^cells/200 ml) | 1 |
| PMBC  control | Makrigiannakis, A.et al.(2019) | Non-RCT | during 2015 | age ≤ 30 ; with a history of at least 3 failed IVF cycles with a cumulative transfer of 6 fresh cleavage stage embryos or 3 fresh blastocyst transfers; no history of clinical pregnancy; FSH<12 mIU/ml at the 3rd day of the menstrual cycle. |  | Two or three embryos at cleavage stage | fresh | one day before embryo transfer | a final concentration of 2×107 cells/200 μL supplemented with 107M CRH | 1 |
| PBMC  control | Nobijari, F.F.et al.(2019) | RCT | 2015 and 2017 | primary infertility and a history of at least three RIF | a history of repeated infectious diseases, endometriosis or uterine disease (such as uterine leiomyomas) or a known aetiology of RIF (such as chromosomal disorders) | cleavage embryo or blastocyst | FET | Two days before embryo transfer | 0.4 ml (2 x 10^7^ cells/200µl) | 1 |
| PBMC  control | Yu, N.et al.(2016) | RCT | September 2013 and May 2014 | All patients had experienced three or more failures of IVFET therapy without poor ovarian reserve (FSH <15 mIU/mL) and were less than 35 years of age | hydrosalpinx, Asherman syndrome, dysontogenesis, endometrial organic disease, chromosomal abnormalities, and severe endometriosis. | cleavage embryo | FET | two day of progesterone administration | 200 μl of PBMC (1–2×10^7^ cells) suspension | 1 |
| PBMC  control | Okitsu, O.et al.(2013) | Prospective cohort study | May 2007 and February 2010 | All patients had experienced one or more failures of IVFET therapy without poor ovarian reserve (FSH <15 mIU/ml). | Patients who had known etiologies of recurrent failures of implantation, such as chromosomal abnormalities, presence of antiphospholipid antibodies, or mutations involving parts of the coagulation system, such as deficiencies of factor XII, protein C, and protein S | Cleavage embryo and blastocyst | FET | On day 1 for embryo transfer at the early cleavage stage or on day 2 for embryo transfer at the blastocyst stage. | 500 ul(3 × 10^7^ cells) | 1 |
| PBMC  control | Madkour, A.et al.(2016) | prospective randomized study | NA | at least two previous failures of implantation after IVF/ ICSI, primary infertility, endometrial thickness < 6 mm in ovulation induction, age < 40 years, regular menstrual cycles, BMI <30, absence of uterine pathology and infectious negative balance. | polycystic ovary syndrome and uterine pathology. | cleavage embryo | fET | 2 days before embryo transfer | 1 × 10^6^ cells in 0.4 ml | 1 |
| PBMC  placebo | Pourmoghadam, Z.et al.(2020) | RCT | October 2017 and September 2018 | at least three previous failures of IVF/ET therapy, primary infertility, age<45 years, regular menstrual cycles, and BMI < 30. | Ovulatory disorder, uterine pathology and anomalies, tubal factors, poor ovarian reserve, chromosomal abnormalities, presence of auto antibodies such as antiTPO, antiTG, ACA, APA, ANA, and antidsDNA | three (early cleavage stage) and day five (blastocyst) | FET | Two days before ET | 500 μl  (1520×10^6^ cells) | 1 |
| PBMC  placebo | Jie Li et al. (2022) | RCT | From June 2019 to December 2021 | The chromosome karyotype of both couples is normal; FSH<15IU/L; age < 40 years old;; 1 or more frozen embryos was in the center; No clinical pregnancy was obtained after ≥2 times embryo transplantation or the cumulative transferred embryos ≧10. | organic disease of uterus; Endometriosis; Polycystic ovary syndrome, thyroid dysfunction, hyperprolactinemia and other endocrine diseases; immune antibodies were abnormal; with hydrosalpinx; Hysteroscopy showed abnormality | Cleavage embryo or blastocysts | FET | 3 days and 1 day before FET | 2×10^6^/ml | 2 |
| PBMC  placebo | Hui Xu et al. (2021) | RCT | June 2018 to June 2020 | ≥ 2 transplant failures with high quality embryo, unknown reasons | with incomplete general data; with poor compliance; with serious systemic diseases, tumors and other diseases; Unilateral or bilateral hydrosalpinx, hydrocele; with uterine malformations and organic diseases of uterus; with endometritis; endometrial tuberculosis or endometriosis. | NA | FET | 1 day before FET | 1 ml (1×10^7^/100μl) | 1 |
| PBMC  control | Lina Lei et al. (2021) | RCT | June 2018 to June 2019 | Consistent with RIF diagnosis; < 40 years old; FSH < 15U/L; The chromosome karyotype of both husband and wife is not abnormal; Patients voluntarily participated in the study and signed informed consent. | Unilateral or bilateral hydrosalpinx, hydrocele; with uterine malformations and organic diseases; with endometritis; with endometrial tuberculosis or endometriosis. | cleavage embryo | FET | 1 day before FET | ２００μｌ | 1 |
| PBMC  control | Huimin Zhao et al. (2020) | RCT | January 2016 to September 2018 | infertility; FSH < 15 U/L; Highquality embryo transfer ≥3 times; Informed consent of both husband and wife. | chromosome karyotype of either spouse of the patient is abnormal; uterus is malformed; The patient was complicated with pelvic organic lesions. | Cleavage embryo | FET | 3 days before FET | NA | 1 |
| PBMC  control | Huakun Zhang et al. (2019) | NonRCT | January 2016 to August 2017 | < 40 years old; at least 2 or more previous transplant cycles, and ≥4 embryos of good quality but clinical pregnancy was not achieved; FSH <15 U/L; at least one highquality embryo was present. | Abnormal chromosome karyotype; Female antiphospholipid antibody positive; the uterus has organic lesions; hydrosalpinx. | cleavage embryo | FET | 3 days before FET | NA | 1 |
| PBMC  control | Yungai Xiang et al. (2019) | RCT | January 2015 to December 2016 | Age < 40 years old. FSH < 15 U/L. The chromosome karyotype of both couples is normal. Clinical pregnancy was not achieved with more than 3 times transplantation or more than 10 embryos. | Uterine malformations and organic lesions; endometrial tuberculosis; heterotopic disease; Unilateral or bilateral hydrosalpinx;  endometritis, uterine effusion. | cleavage embryo | FET | one days before FET | 200μL (2×10^6^/mL) | 1 |
| PBMC  control | Xue Lu et al. (2019) | RCT | September 2018 to February 2019 | < 40 years old;Rule out poor ovarian reserve function (basic FSH < 10IU/L); 3 or more times of transplantation | Hydrosalpinx; endometrial polyps; uterine gland diseases; Chromosomal abnormalities; Uterine fibroids; organic lesions of endometrial; Uterine deformity. | NA | FET | On the day of progesterone administration | NA | 1 |
| PBMC  control | Fang Cao et al. (2015) | Nonrandomized controlled trial | January 2014 January 2015 | have transplanted high quality embryos at cleavage stage 3 times or more; The endometrial Salle score of the day before prior cycle embryo transfer (ET) was ≥13; Basal FSH < 15 U/L; (3) The chromosome karyotype of both couples is normal. | Patient has uterine malformations, endometriosis and other pelvic organic diseases. | Cleavage embryo | FET | 3 days before FET | NA | 1 |
| PBMC  control | Qi Lin et al. (2013) | RCT | March 2010 to December 2012 | After 2 to 6 egg retrieval cycles and transfer of more than 10 highquality embryos without obtaining clinical pregnancy, or after more than 3 retrieval cycles and transfer of highquality embryos in each cycle without obtaining clinical pregnancy. | The chromosomal karyotype was abnormal; the female had positive antiphospholipid antibody, with organic lesions in uterus; with hydrosalpinx. | Cleavage embryo | FET | 3 days before FET | NA | 1 |
| ECS  control | Bing, L.et al.(2020) | Nonr-RCT | March 2019 December 2019 | Age ≤35 years old; Regular menstrual cycle, BMI 19 ~ 24; basal  FSH < 10 IU/L, no hormone drugs have been used in the past 3 months | the shape of the uterine cavity is abnormal; with endometrial polyps, endometriosis or adenomyosis of the uterus, with endocrine dysfunction and systemic diseases. | NA | FET | 4 days and 1 day before FET | NA | 2 |
| G-CSF  Control  placebo | DavariTanha, F.et al.(2016) | RCT | December 2011 until January 2014 | all patients with RIF under the age of 40 years old | Women with history of renal disease, sickle cell disease or malignancy or sensitivity of GCSF | Cleavage embryo and blastocyst | fET and FET | the day of oocyte puncture or day of progesterone administration of FET cycle | 1 ml (300µg/1ml) | 1 |
| G-CSF  placebo | Kalem, Z.et al.(2020) | prospective randomized controlled trial | March 2016 and December 2017 | Women under the age of 40 who met the RIF definition, basal FSH <15 IU/mL | with autoimmune diseases, with congenital uterine anomalies, with Asherman’s syndrome, with uterine cavity distorted by myoma or endometrial polyps, with confirmed endometriosis or endometrioma, and patients for whom GCSF was contraindicated  endometrial thickness less than 7 mm (measured in the pretreatment midcycle) | Cleavage embryo or blastocyte | fET | once a day on hCG day, before hCG injection | 1mL  (30 mIU) | 2 |
| G-CSF  Contro  placebo | Huang, P.et al.(2022) | RCT |  | primary infertility, two failed implantations (each time containing at least one highquality embryo), ≤38 years old;1≤BMI≤24, normal endometrial thickness (8–16mm), two or more frozen embryos available, including at least one goodquality embryo or one goodquality blastocyst. | other gynaecological conditions, such as endometrial polyps, intrauterine adhesions, or uterine submucosal myomas, chronic endometritis that might cause endometrial abnormalities; adenomyosis; hydropic Fallopian tubes; polycystic ovarian syndrome; or stage III or higher endometriosis. | Cleavage embryo or blastocyte | FET | three days before the ET | 1 ml (150 mg) | 1 |
| G-CSF  control | Eftekhar, M.et al.(2016) | RCT | October 2014 and February 2015 | Women between 2040 years old with history two implantation failures | Participants with sickle cell disease, chronic neutropenia, malignancy history, renal failure, congenital fructose intolerance, respiratory infection, endometriosis and sever male factor | Cleavage embryo | fET | after ovarian puncture | 0.5 ml (300 µg/ml) | 1 |
| G-CSF  placebo | Karimi, A.et al.(2020) | RCT | December 2015 and September 2018 | . All patients with at least two pervious unsuccessful IVF/ICSI cycles, unexplained RIF patients | Patients older than 38 years old, those with BMI>30, FBS>110, FSH>12, congenital or acquired uterine anomaly, systemic disease, uncontrolled diabetes, Hypertension, thyroid, renal disease, TPO>500, abnormal karyotype, azoospermia with negative sperm in biopsy and ongoing cancer | Cleavage embryo | fET | just after ovarian puncture | 0.5 ml  (300ugr) | 1 |
| G-CSF  placebo | Tingting Xia et al. (2022) | RCT | January 2019 to September 2020 | Age < 40 years old, Implantation failure with three or more transplant cycles or four to six highscoring cleavage stage embryos or three or more highscoring blastocysts | After screening, there may be uterine factors, gamete and embryo abnormalities, immune and endocrine functions, transplantation techniques and psychological factors, with a history of allergy to GCSF; refused to participate in the researcher with full informed consent. | 1 to 2 high quality cleavage embryos | FET | Days 9 and 12 after estrogen use | 1.2 ml  (300ug) | 2 |
| GCSF+AXaIUsc  control | Zengrong Tu et al. (2020) | RCT | January 2016 to June 2018 | The thickness of the thickest part of the endometrium measured by vaginal Bultrasound after injection of human chorionic gonadotropin (HCG) is ≤7 mm, IVFET or freeze thaw embryo transfer (FET) ≥3 times, and the number of highquality embryos transferred ≥4, but no pregnancy achieved. | Intrauterine adhesions; submucous uterine fibroids; endometrial polyps and other intrauterine spaceoccupying lesions and uterine malformations; Endocrine related diseases, such as hyperprolactinemia, thyroid dysfunction, etc. | 1 to 2 highquality embryos | fET | From the 8th day of the menstrual cycle, once every other day, to the last time on the day of egg retrieval | 0.9 ml (150μg/) | 3～5 |
| G-CSF  control | Xiqian Zhang et al. (2020) | RCT | March 2016 to April 2019 | Age: 2039 years old female, RIF patients; | Male, female and both chromosome abnormal patients; Abnormal uterine anatomy; the uterus and uterine cavity have organic lesions and uterine adhesion; with chronic diseases such as hypertension and diabetes; Endometriosis; Abnormal thin endometrium (≤7 mm); untreated endometritis; Prethrombotic state. | Cleavage embryo | FET | First: Late follicle natural cycle or HRT artificial cycle supplemented estrogen on day 12；Second: Ovulation day of natural cycle or endometrial transformation day of HRT artificial cycle | 150μg | 2 |
| G-CSF  control | Jun He et al. (2015) | RCT | March 2013 to June 2014 | RIF; Hysteroscopy was performed to rule out endometrial lesions and uterine abnormalities, and chromosomal abnormalities or medical diseases and other causes of embryo implantation failure were ruled out. | too old (≥40 years old), do not have good quality embryos, or those who cancel the transplant cycle because the endometrium is ≤6mm | Cleavage embryo | fET or FET | the day of OPU or 3 days before FET | 1 ml  (150mg/ml) | 1 |
| PRP  control | Nazari, L.et al.(2020) | RCT | Between 2016 and 2017 | age below 40 years and body mass index (BMI) below 30 kg/m2 | uterine abnormalities, hormonal disorders, immunological and haematological disorders, azoospermia, testicular sperm extraction or aspiration, anatomical disorders of the male genital tract, varicocele and chromosomal abnormalities in the couples | Good quality blastocysts | FET | 48h before blastocyst transfer | 0.5 ml  (contained platelets at 4–5 times higher concentration than peripheral blood) | 1 |
| PRP  control | Zamaniyan, M.et al.(2021) | a blind randomized clinical trial | February 2016 to January 2019 | age between 20–40 years, Body Mass Index (BMI) under 30 kg/m2, and normal hysterosalpingography. | hematologic disorders, immunologic disorders, hormonal disorders, chromosomal and genetic anomalies, and renal failure. | one or two good quality blastocysts | FET | 2 days before embryo transfer | 0.5 ml  (4–6 times higher concentration than peripheral blood) | 1 |
| PRP  placebo | Bakhsh, A.S.et al.(2022) | RCT | NA | infertile women with a history of RIF; age below 40 years; and body mass index (BMI) below 30kg/m2 . | hematological and immunological disorders; cancers; hormonal disorders; Hb<11g/dl and PLT<150000mm3; chromosomal and genetic abnormalities; taking anticoagulants; taking NSAIDs for 7 days prior to the procedure; smoking; uterine abnormalities; FSH <12IU; uncontrolled underlying diseases such as diabetes, and hypertension; simultaneous administration of other drugs (prednisolone, IVIG, GCSF); and simultaneous endometrial scratch. | good quality embryos | FET | 2 days before blastocyst transfer | 0.5CC, contained platelet 45 times more than a peripheral blood sample | 1 |
| PRP  control | Nazari, L.et al.(2021) | RCT | between 2018 and 2020 | All of the women had a history of failure to achieve pregnancy after three or more embryo transfers with highquality embryos. Age between 18 and 38, BMI ≤30 kg/m2, basal serum FSH level ≤10 mIU/ml | PCOS, OHSS, endometriosis, presence of spaceoccupying lesions, history of miscarriage or ectopic pregnancy, myomas, polyps, adhesions, previous pelvic surgeries, failed fertilization, and less than two embryos available for transfer. participats with severe male factor of their spouses and chromosomal abnormalities | 1–2 blastocysts | FET | 48 h before embryo transfer | 0.5 ml | 1 |
| PRP  control | Aghajanzadeh, F.et al.(2020) | NonRCT | June 2017 to August 2018 | healthy women aged 1840 years and a history of four or more episodes of implantation failure with a BMI <30 Kg/m2 | Women with hemoglobin disorders, immune or endocrine disorders, genetic or chromosomal abnormalities, uterine infection or anomaly, history of uterine incisions, fever, malignancy, females on NSAIDs 10 days prior to the intervention or on corticosteroids over the past month, smokers, and women with the medium or poorquality frozenthawed embryos | two good quality embryos | frozenthawed embryo transfers | 48 hours prior to embryo transfer. |  | 1 or 2 |
| PRP  control | Noushin, M.A.et al.(2021) | Prospective observational cohort study. | March 2019 to May 2020 | Women aged <40 years with a history of RIF undergoing frozen embryo transfer (FET) | women with BMI >30 kg/m2, congenital and untreated acquired uterine abnormalities, untreated hydrosalpinges, poor ovarian responder as per the Bologna criteria, thrombophilia, or uncontrolled endocrine or hematologic dysfunction; those undergoing preimplantation genetic testing cycles; and those who had thin endometrium (<8 mm) in the index FET cycle Severe male factor infertility, difficult ET, only poorquality embryos available, and couple with genetic and chromosomal abnormalities | Two embryos of day 3 developmental stage with at least one morphologically goodquality or grade 1 embryo | FET | during the index FET cycle when the endometrium was approximately 7 mm | 1 mL | 1 |
| PRP  control | Tehraninejad, E.S.et al.(2021) | NonRCT | between 2016 and 2018 | RIF | age ≥35 years, endometrial thickness <7 mm, basal FSH levels>10 mIU/mL, severe male factor such as azoospermia, intrauterine disorders, thrombophilia, thyroid dysfunction, positive antiphospholipid antibodies or chromosomal abnormality in a couple. | two good to moderate quality blastocysts | FET | 2 days before the FET | 1 mL | 1 |
| PRP  control | Baybordi, E.et al.(2022) | RCT | May 2017 to December 2019 | women of childbearing age with a history of RIF during ART treatments. | women aged > 18 and aged < 45 years old, diagnosed cancers, anemia, platelets count <150,000/cc, pregnancy, use if anticoagulants, use of nonsteroidal antiinflammatory drugs up to 10 days before the procedure, any physical or mental illness that affects the participant’s immunity and admission and disrupt the process of implantation and participants followup. | blastocyst | FET | day 10th of the HRT cycle | 0.51 ml | repeated as needed  (once or twice per cycle) |
| PRP  control | Salehpour, L.N.S.et al.(2020) | RCT | Between 2016 and 2017 | Failed to conceive after 3 or more embryo transfers with highquality embryos and candidates for frozenthawed embryo transfer (FET), age below 40 years and  BMI below 30 kg/m2 | uterine abnormalities, hormonal disorders, immunological and haematological disorders, azoospermia, testicular sperm extraction or aspiration, anatomical disorders of the male genital tract, varicocele and chromosomal abnormalities in the couples | Good quality blastocysts | FET | 2 dayys before blastocyst transfer | 0.5 ml | 1 |
| PRP  control | Zargar, M.et al.(2021) | RCT | NA | infertile women with at least two IVF failures and age below 41 years old | women with chromosomal, genetic, and uterine abnormalities, hematological or immunological disorders, and hormonal disorders; also, the embryos that arise from such maternal and paternal abnormalities were excluded | NA | FET | 2 days before embryo transfer and after | PRP  (1.5 mL) | once or twice |
| PRP  control | Kusumi, M.et al.(2020) | RCT | February 2018 through January 2019 | age of 2050 years, oocyte retrieval performed at age 42 years or younger, and endometrial thickness of ≤7 mm. | hepatic disorders, hemoglobin level of <11 g/dL, platelet count of <150 000/mm3, use of anticoagulants, and pregnancy | NA | FET | 10th and 12th days of the second HRT cycle | 1 mL | 2 |
| PRP  control | Ershadi, S.et al.(2022) | NonRCT | 2019 | age under 40 years and a history of two to three IVF failures | any uterine anomalies, having an underlying disease, taking any specific medication, not having proper fetus for transfer on the day of ET, insufficient endometrial thickness for ET. | goodquality blastocyte | FET | 2 days before the ET | 0.5 mL | 1 |
| PRP  control | Kepeng Liu et al. (2021) | RCT | June 2018 to July 2019 | No pregnancy has occurred after 3 or more transfers of good quality cleavage embryos, age<40 years old, BMI< 30kg/m2, at least one good quality embryo can be obtained based on morphological assessment. | Abnormalities of the woman's uterus, hormonal abnormalities, chromosomal abnormalities in one of the spouses, immunological and hematological abnormalities, abnormalities caused by factors in the husband. | At least 1 good quality cleavage embryo | FET | 2 days before FET | 1ml | 1 |
| PRP  control | Xiaoling Hu et al. (2020) | RCT | August 2018 August 2019 | Age ≤40 years old; Infertility factors mainly include: male factors, ovulation disorders, pelvic fallopian tube factors; Embryo implantation failed twice or more. | NA | NA | FET | 5 days to 3 days before endometrial luteal transformation | 1ml | 13 |
| PRP  control | Xiaoling Hu et al. (2019) | RCT | December 2017 to July 2019 | The patient is less than 40 years old; The number of embryo transfer is greater than or equal to 2 times; no organic diseases of pelvic organs. | intrauterine adhesion; adenomyopathy; Patients with uterine fibroids; Hydrosalpinx; Not cooperating with researchers | Cleavage embryo | FET | 3 days before progesterone administration | NA | 1 |
| PRP+G-CSFsc | Dieamant, F.et al.(2019) | NonRCT | February 2017 to October 2017 | ≥2embryo transfers and at least 5 morphologically good embryos transferred,a normal karyotype for her and her partner. | no evidence of uterine defects, ultrasonographic evidence of hydrosalpinx, infections, endocrine problems, coagulation defects, thrombophilia and autoimmune defects. | NA | fET | 2 days before the ET | 0.7ml | 1 |
| PRP+G-CSF | Ning Ma et al. (2023) | NonRCT | January 2020 to July 2022 | Basal FSH < 10 U/L; No hydrosalpinx or ligation; Age < 40 years old, have received ≥3 embryo transfer cycles, and a cumulative transfer of ≥4 highquality embryos without clinical pregnancy | patients with pelvic tumor; Having contraindications of pregnancy or suffering from diseases that have definite effects on pregnancy; Abnormal anatomical structure of uterus; Endocrine examination was abnormal; Autoimmune antibodies positive. | Cleavage embryo or blastocysts | FET | before the embryo transplantation | 0.9mL (PRP+150μgGCSF) | 1 |
| HCG  G-CSF | Bakry, M.S.et al.(2022) | RCT | between 10th October 2020 and 20th December 2020 | women aged 20–43 years with a history of RIF who undergoing ICSI cycles. | Women with associated medical disorders were excluded as diabetes, hypertension, heart diseases, and thyroid disorders. | one to three grade A blastocyte | fET | on embryo transfer day | 500 IU  (every one cm contained 500 IU HCG. 300 mcg/ 1.0 GCSF | 1 |
| placebo  HCG  G-CSF | Torky, H.et al.(2022) | RCT | January 2019 and January 2020 | aged between 20 and 39 years old, undergoing IVF/ICSI who had a history of recurrent implantation failure | women with hypersensitivity or at high risk of developing complications from any of the used medications, with sickle cell nephropathy, with a history of malignancy, with poor quality embryos, and women at risk of OHSS | one to three gradeA embryos | fET | Ovum Pick Up Day | 1 cc containing 100 µg GCSF;  1 cc contained 500 IU of hCG | 1 |
| CONTROL PBMC HCG | Mei, J.et al.(2022) | NonRCT | January 2016 and December 2020 | All patients had previously experienced at least one failure of IVFET therapy, and received FET in Reproductive Medicine Center of Nanjing Drum Tower hospital | Patients who had old age (age >35 years old), poor ovarian reserve, thin endometrium r, congenital anatomical abnormalities, chromosomal abnormalities for PGTA, presence of antiphospholipid antibodies, or mutations of the coagulation system | blastocyte | FET | 2 days before FET | 700 μl (PBMC 1×10^7^ cells; 83ug rHCG | 1 |
| CONTROL PBMC HCG GCSF placebo | Dujuan Xu et al. (2023) | RCT | January 2020 December 2021 | Clinical pregnancy is not achieved after transferring more than 4 highquality embryos in at least 3 fresh or frozen cycles; Age 1840 years old; Good mental state, can cooperate with this study. | Hysteroscopy showed abnormal uterine cavity morphology, complicated with uterine adhesions, endometrial polyps or uterine submucous myoma; chromosomal abnormalities, active infectious diseases; Hydrosalpinx and weak ovarian reserve function; Combined with cardiovascular system, respiratory system, digestive system and other systemic diseases; Combined with hypothyroidism, hyperthyroidism or thrombotic disease. | High quality embeyo | FET | 3 days before FET | 1mL  (1000 IU/ml hcg, PBMCs (1~5) × 10^6^ /mL, GCSF 1 mL) | 1 |
| Control GH G-CSF | Zhijin Hou et al. (2022) | RCT | September 2019 to January 2021 | Age≤40 years old; Patients with≥2 times embryo transfers or with cumulative 4 highquality embryos, but still have not obtained pregnancy | Suffering from malformation of reproductive system, abnormal development of reproductive system, acute and chronic pelvic inflammatory disease, etc. Diagnosed with autoimmune diseases; Patients with contraindications for GCSF or GH use. | Cleavage or blastocyte | FET | Days 10, 12 and 14 of menstruation | GCSF  (1.5 ml); GH (1 g, Soluble in 1.5ml 0.9% sodium chloride solution) | 3 |
| Control placebo HCG G-CSF PBMC | Pingxiu Huang et al. (2018) | RCT | NA | Transplantation failure ≥2 times (each transplantation contains highquality embryos); ≤ 38 years old, 18≤BMI≤ 24, normal intimal thickness, and had two or more D3day embryos (of which at least one good quality embryo) or one good quality blastocyst cryopreserved. | obvious intrauterine adhesions, uterine malformations, hydrosalpinx, or decreased ovarian reserve. | Cleavage or blastocyte | FET | 2 or 3 days before FET | 1ml；hCG  (1000IU), GCSF  (300ng/ml); PBMC：1.0ml | 1 |
