# Supplementary material for: Uterine infusion strategies for infertile patients with recurrent implantation failure: a systematic review and network meta-analysis
Source: Reprod Biol Endocrinol. 2024 Apr 16;22:44. doi: 10.1186/s12958-024-01221-x (PMC11020641; doi:10.1186/s12958-024-01221-x)
Supplement: Supplementary file 1 — Additional file 1: Figure S1. Risk of bias assessment. a. Risk of bias summary; b. Risk of bias graph. Figure S2. Forest plot of the live birth in direct pair-wise meta-analysis. Figure S3. Network plots of eligible comparisons for secondary outcomes: clinical pregnancy rate. a. Live birth; b. Embryo implantation; c. Chemical pregnancy; d. Miscarriage. Figure S4. Forest plot of the embryo implantation in direct pair-wise meta-analysis. Figure S5. Forest plot of the chemical pregnancy in direct pair-wise meta-analysis. Figure S6. Forest plot of the miscarriage in direct pair-wise meta-analysis. Figure S7. Funnel plot of the pregnancy outcomes. Figure S8. Subgroup analysis of forest plot of the clinical pregnancy in the direct pair-wise meta-analysis by English researches. Figure S9. Subgroup analysis of forest plot of the clinical pregnancy in the direct pair-wise meta-analysis by Chinese researches. Supplemental Table S1. Characteristics of studies included in meta-analyses. Supplemental Table S2. Risk of bias assessment of the other prospective studies. Supplemental Table S3. Network meta-analysis for live birth comparing diverse uterine infusion strategies. Supplemental Table S4. Network meta-analysis for implantation comparing diverse uterine infusion strategies. Supplemental Table S5. Network meta-analysis for chemical pregnancy comparing diverse uterine infusion strategies. Supplemental Table S6. Network meta-analysis for miscarriage comparing diverse uterine infusion strategies. Supplemental Table S7. Subgroup analysis of network meta-analysis for clinical pregnancy by English researches. Supplemental Table S8. Subgroup analysis of network meta-analysis for clinical pregnancy by Chinese researches. [file 12958_2024_1221_MOESM1_ESM.zip › Table S3 live birth.docx]

**Table S3** Network meta-analysis for live birth comparing diverse uterine infusion strategies.

| **Groups/pregnant outcomes** | **GCSF** | **GH** | **HCG** | **PBMC** | **PRP** | **PRP+G-CSFsc** | **Placebo** |
| --- | --- | --- | --- | --- | --- | --- | --- |
| **Control** | 2.32 (0.85, 6.20) | 2.07 (0.38, 12.00) | 2.56 (0.72, 9.34) | 1.89 (0.77, 4.70) | 4.85 (1.76, 14.56) | 0.99 (0.14, 7.13) | 2.31 (0.83, 6.62) |
| **GCSF** |  | 0.91 (0.17, 5.18) | 1.11 (0.27, 4.74) | 0.82 (0.25, 2.73) | 2.10 (0.52, 9.08) | 0.43 (0.05, 3.97) | 1.00 (0.38, 2.61) |
| **GH** |  |  | 1.22 (0.14, 9.60) | 0.90 (0.13, 6.09) | 2.31 (0.30, 17.52) | 0.47 (0.03, 6.28) | 1.09 (0.17, 7.24) |
| **HCG** |  |  |  | 0.74 (0.20, 2.74) | 1.90 (0.35, 10.39) | 0.39 (0.04, 4.12) | 0.91 (0.26, 3.21) |
| **PBMC** |  |  |  |  | 2.58 (0.65, 10.26) | 0.52 (0.06, 4.73) | 1.23 (0.39, 3.88) |
| **PRP** |  |  |  |  |  | 0.20 (0.02, 1.94) | 0.47 (0.10, 2.06) |
| **PRP+G-CSFsc** |  |  |  |  |  |  | 2.31 (0.25, 22.52) |
