# Supplementary material for: Uterine infusion strategies for infertile patients with recurrent implantation failure: a systematic review and network meta-analysis
Source: Reprod Biol Endocrinol. 2024 Apr 16;22:44. doi: 10.1186/s12958-024-01221-x (PMC11020641; doi:10.1186/s12958-024-01221-x)
Supplement: Supplementary file 1 — Additional file 1: Figure S1. Risk of bias assessment. a. Risk of bias summary; b. Risk of bias graph. Figure S2. Forest plot of the live birth in direct pair-wise meta-analysis. Figure S3. Network plots of eligible comparisons for secondary outcomes: clinical pregnancy rate. a. Live birth; b. Embryo implantation; c. Chemical pregnancy; d. Miscarriage. Figure S4. Forest plot of the embryo implantation in direct pair-wise meta-analysis. Figure S5. Forest plot of the chemical pregnancy in direct pair-wise meta-analysis. Figure S6. Forest plot of the miscarriage in direct pair-wise meta-analysis. Figure S7. Funnel plot of the pregnancy outcomes. Figure S8. Subgroup analysis of forest plot of the clinical pregnancy in the direct pair-wise meta-analysis by English researches. Figure S9. Subgroup analysis of forest plot of the clinical pregnancy in the direct pair-wise meta-analysis by Chinese researches. Supplemental Table S1. Characteristics of studies included in meta-analyses. Supplemental Table S2. Risk of bias assessment of the other prospective studies. Supplemental Table S3. Network meta-analysis for live birth comparing diverse uterine infusion strategies. Supplemental Table S4. Network meta-analysis for implantation comparing diverse uterine infusion strategies. Supplemental Table S5. Network meta-analysis for chemical pregnancy comparing diverse uterine infusion strategies. Supplemental Table S6. Network meta-analysis for miscarriage comparing diverse uterine infusion strategies. Supplemental Table S7. Subgroup analysis of network meta-analysis for clinical pregnancy by English researches. Supplemental Table S8. Subgroup analysis of network meta-analysis for clinical pregnancy by Chinese researches. [file 12958_2024_1221_MOESM1_ESM.zip › Table S4 implantation.docx]

**Table S4** Network meta-analysis for implantation comparing diverse uterine infusion strategies.

| **Groups/pregnant outcomes** | **DEX** | **ECS** | **GCSF** | **G-CSF+AXaIUsc** | **GH** | **HCG** | **PBMC** | **PRP** | **PRP+G-CSF** | **PRP+G-CSFsc** | **Placebo** |
| --- | --- | --- | --- | --- | --- | --- | --- | --- | --- | --- | --- |
| **Control** | 2.32 (0.94, 5.84) | 1.63 (0.72, 3.77) | 2.62 (1.89, 3.74) | 2.81 (1.20, 6.46) | 3.57 (1.53, 8.25) | 1.94 (1.33, 2.79) | 3.03 (2.25, 4.32) | 2.77 (1.55, 4.83) | 2.45 (0.90, 7.09) | 1.03 (0.34, 3.17) | 1.41 (0.93, 2.14) |
| **DEX** |  | 0.70 (0.20, 2.37) | 1.13 (0.43, 2.98) | 1.20 (0.35, 4.15) | 1.54 (0.44, 5.17) | 0.83 (0.31, 2.22) | 1.30 (0.51, 3.43) | 1.18 (0.41, 3.43) | 1.06 (0.27, 4.20) | 0.45 (0.11, 1.84) | 0.61 (0.22, 1.63) |
| **ECS** |  |  | 1.61 (0.65, 3.94) | 1.72 (0.54, 5.61) | 2.20 (0.67, 7.47) | 1.19 (0.49, 2.89) | 1.85 (0.80, 4.63) | 1.70 (0.62, 4.74) | 1.52 (0.43, 5.61) | 0.64 (0.16, 2.61) | 0.87 (0.34, 2.18) |
| **GCSF** |  |  |  | 1.07 (0.46, 2.42) | 1.36 (0.58, 3.11) | 0.74 (0.50, 1.08) | 1.15 (0.76, 1.79) | 1.05 (0.54, 2.00) | 0.94 (0.32, 2.84) | 0.39 (0.12, 1.28) | 0.54 (0.35, 0.82) |
| **G-CSF+AXaIUsc** |  |  |  |  | 1.28 (0.39, 4.01) | 0.69 (0.29, 1.65) | 1.08 (0.46, 2.68) | 0.97 (0.35, 2.69) | 0.88 (0.24, 3.31) | 0.37 (0.09, 1.44) | 0.50 (0.21, 1.26) |
| **GH** |  |  |  |  |  | 0.54 (0.23, 1.32) | 0.85 (0.36, 2.10) | 0.77 (0.28, 2.13) | 0.70 (0.19, 2.60) | 0.29 (0.07, 1.15) | 0.39 (0.16, 0.99) |
| **HCG** |  |  |  |  |  |  | 1.57 (1.05, 2.42) | 1.43 (0.73, 2.79) | 1.27 (0.44, 3.82) | 0.53 (0.17, 1.72) | 0.73 (0.50, 1.07) |
| **PBMC** |  |  |  |  |  |  |  | 0.91 (0.46, 1.70) | 0.81 (0.27, 2.37) | 0.34 (0.11, 1.08) | 0.47 (0.30, 0.70) |
| **PRP** |  |  |  |  |  |  |  |  | 0.89 (0.28, 3.01) | 0.38 (0.11, 1.32) | 0.51 (0.25, 1.02) |
| **PRP+G-CSF** |  |  |  |  |  |  |  |  |  | 0.42 (0.09, 1.85) | 0.57 (0.19, 1.72) |
| **PRP+G-CSFsc** |  |  |  |  |  |  |  |  |  |  | 1.38 (0.40, 4.47) |
