# Supplementary material for: Uterine infusion strategies for infertile patients with recurrent implantation failure: a systematic review and network meta-analysis
Source: Reprod Biol Endocrinol. 2024 Apr 16;22:44. doi: 10.1186/s12958-024-01221-x (PMC11020641; doi:10.1186/s12958-024-01221-x)
Supplement: Supplementary file 1 — Additional file 1: Figure S1. Risk of bias assessment. a. Risk of bias summary; b. Risk of bias graph. Figure S2. Forest plot of the live birth in direct pair-wise meta-analysis. Figure S3. Network plots of eligible comparisons for secondary outcomes: clinical pregnancy rate. a. Live birth; b. Embryo implantation; c. Chemical pregnancy; d. Miscarriage. Figure S4. Forest plot of the embryo implantation in direct pair-wise meta-analysis. Figure S5. Forest plot of the chemical pregnancy in direct pair-wise meta-analysis. Figure S6. Forest plot of the miscarriage in direct pair-wise meta-analysis. Figure S7. Funnel plot of the pregnancy outcomes. Figure S8. Subgroup analysis of forest plot of the clinical pregnancy in the direct pair-wise meta-analysis by English researches. Figure S9. Subgroup analysis of forest plot of the clinical pregnancy in the direct pair-wise meta-analysis by Chinese researches. Supplemental Table S1. Characteristics of studies included in meta-analyses. Supplemental Table S2. Risk of bias assessment of the other prospective studies. Supplemental Table S3. Network meta-analysis for live birth comparing diverse uterine infusion strategies. Supplemental Table S4. Network meta-analysis for implantation comparing diverse uterine infusion strategies. Supplemental Table S5. Network meta-analysis for chemical pregnancy comparing diverse uterine infusion strategies. Supplemental Table S6. Network meta-analysis for miscarriage comparing diverse uterine infusion strategies. Supplemental Table S7. Subgroup analysis of network meta-analysis for clinical pregnancy by English researches. Supplemental Table S8. Subgroup analysis of network meta-analysis for clinical pregnancy by Chinese researches. [file 12958_2024_1221_MOESM1_ESM.zip › Table S5 Chemical pregnancy.docx]

**Table S5** Network meta-analysis for chemical pregnancy comparing diverse uterine infusion strategies.

| **Groups/pregnant outcomes** | **G-CSF** | **GH** | **HCG** | **PBMC** | **PRP** | **PRP+G-CSF** | **Placebo** |
| --- | --- | --- | --- | --- | --- | --- | --- |
| **Control** | 1.85 (0.69, 4.81) | 1.57 (0.20, 11.53) | 1.59 (0.54, 4.70) | 1.31 (0.07, 22.26) | 1.90 (1.00, 3.54) | 3.45 (0.48, 26.19) | 1.49 (0.50, 4.53) |
| **G-CSF** |  | 0.84 (0.12, 6.41) | 0.87 (0.30, 2.51) | 0.72 (0.03, 14.57) | 1.02 (0.34, 3.36) | 1.89 (0.21, 17.83) | 0.81 (0.33, 2.07) |
| **GH** |  |  | 1.01 (0.12, 9.01) | 0.83 (0.03, 28.40) | 1.20 (0.16, 10.27) | 2.23 (0.14, 36.45) | 0.94 (0.11, 8.33) |
| **HCG** |  |  |  | 0.82 (0.04, 17.49) | 1.19 (0.35, 4.11) | 2.20 (0.22, 22.50) | 0.93 (0.30, 2.98) |
| **PBMC** |  |  |  |  | 1.46 (0.08, 27.97) | 2.71 (0.08, 94.46) | 1.14 (0.05, 24.65) |
| **PRP** |  |  |  |  |  | 1.83 (0.23, 15.20) | 0.79 (0.22, 2.83) |
| **PRP+G-CSF** |  |  |  |  |  |  | 0.42 (0.04, 4.13) |
