# Supplementary material for: Uterine infusion strategies for infertile patients with recurrent implantation failure: a systematic review and network meta-analysis
Source: Reprod Biol Endocrinol. 2024 Apr 16;22:44. doi: 10.1186/s12958-024-01221-x (PMC11020641; doi:10.1186/s12958-024-01221-x)
Supplement: Supplementary file 1 — Additional file 1: Figure S1. Risk of bias assessment. a. Risk of bias summary; b. Risk of bias graph. Figure S2. Forest plot of the live birth in direct pair-wise meta-analysis. Figure S3. Network plots of eligible comparisons for secondary outcomes: clinical pregnancy rate. a. Live birth; b. Embryo implantation; c. Chemical pregnancy; d. Miscarriage. Figure S4. Forest plot of the embryo implantation in direct pair-wise meta-analysis. Figure S5. Forest plot of the chemical pregnancy in direct pair-wise meta-analysis. Figure S6. Forest plot of the miscarriage in direct pair-wise meta-analysis. Figure S7. Funnel plot of the pregnancy outcomes. Figure S8. Subgroup analysis of forest plot of the clinical pregnancy in the direct pair-wise meta-analysis by English researches. Figure S9. Subgroup analysis of forest plot of the clinical pregnancy in the direct pair-wise meta-analysis by Chinese researches. Supplemental Table S1. Characteristics of studies included in meta-analyses. Supplemental Table S2. Risk of bias assessment of the other prospective studies. Supplemental Table S3. Network meta-analysis for live birth comparing diverse uterine infusion strategies. Supplemental Table S4. Network meta-analysis for implantation comparing diverse uterine infusion strategies. Supplemental Table S5. Network meta-analysis for chemical pregnancy comparing diverse uterine infusion strategies. Supplemental Table S6. Network meta-analysis for miscarriage comparing diverse uterine infusion strategies. Supplemental Table S7. Subgroup analysis of network meta-analysis for clinical pregnancy by English researches. Supplemental Table S8. Subgroup analysis of network meta-analysis for clinical pregnancy by Chinese researches. [file 12958_2024_1221_MOESM1_ESM.zip › Table S6 Miscarriage.docx]

**Table S6** Network meta-analysis for miscarriage comparing diverse uterine infusion strategies.

| **Groups/pregnant outcomes** | **DEX** | **ECS** | **G-CSF** | **G-CSF+AXaIUsc** | **GH** | **HCG** | **PBMC** | **PRP** | **PRP+G-CSFsc** | **Placebo** |
| --- | --- | --- | --- | --- | --- | --- | --- | --- | --- | --- |
| **Control** | 9.77*10^5^ (1.62, 3.68*10^21^) | 0.87 (0.18, 3.44) | 0.29 (0.14, 0.64) | 0.18 (0.02, 1.41) | 1.08 (0.13, 6.55) | 0.60 (0.32, 1.19) | 0.38 (0.19, 0.83) | 0.96 (0.44, 1.87) | 3.61 (0.30, 203.06) | 1.10 (0.62, 2.26) |
| **DEX** |  | 0.00 (0.00, 0.57) | 0.00 (0.00, 0.20) | 0.00 (0.00, 0.18) | 0.00 (0.00, 1.01) | 0.00 (0.00, 0.42) | 0.00 (0.00, 0.26) | 0.00 (0.00, 0.62) | 0.00 (0.00, 12.40) | 0.00 (0.00, 0.80) |
| **ECS** |  |  | 0.33 (0.07, 2.05) | 0.22 (0.01, 2.49) | 1.19 (0.09, 13.11) | 0.69 (0.16, 4.09) | 0.45 (0.10, 2.60) | 1.06 (0.22, 6.01) | 4.35 (0.24, 313.90) | 1.31 (0.29, 7.89) |
| **G-CSF** |  |  |  | 0.62 (0.06, 5.00) | 3.65 (0.40, 25.23) | 2.05 (0.93, 4.84) | 1.30 (0.56, 3.34) | 3.36 (1.03, 8.73) | 12.17 (0.85, 821.59) | 3.79 (1.92, 8.25) |
| **G-CSF+AXaIUsc** |  |  |  |  | 5.41 (0.32, 119.79) | 3.40 (0.38, 35.60) | 2.21 (0.24, 23.97) | 5.15 (0.63, 57.71) | 22.88 (0.96, 2991.21) | 6.40 (0.67, 67.29) |
| **GH** |  |  |  |  |  | 0.57 (0.09, 5.25) | 0.36 (0.05, 3.57) | 0.85 (0.13, 8.09) | 3.82 (0.16, 371.18) | 1.07 (0.18, 9.70) |
| **HCG** |  |  |  |  |  |  | 0.63 (0.30, 1.39) | 1.58 (0.55, 3.83) | 5.81 (0.43, 349.84) | 1.79 (1.03, 3.50) |
| **PBMC** |  |  |  |  |  |  |  | 2.48 (0.82, 6.27) | 8.98 (0.64, 638.95) | 2.82 (1.40, 6.10) |
| **PRP** |  |  |  |  |  |  |  |  | 3.96 (0.29, 225.00) | 1.15 (0.50, 3.48) |
| **PRP+G-CSFsc** |  |  |  |  |  |  |  |  |  | 0.31 (0.00, 4.41) |
