# Supplementary material for: Uterine infusion strategies for infertile patients with recurrent implantation failure: a systematic review and network meta-analysis
Source: Reprod Biol Endocrinol. 2024 Apr 16;22:44. doi: 10.1186/s12958-024-01221-x (PMC11020641; doi:10.1186/s12958-024-01221-x)
Supplement: Supplementary file 1 — Additional file 1: Figure S1. Risk of bias assessment. a. Risk of bias summary; b. Risk of bias graph. Figure S2. Forest plot of the live birth in direct pair-wise meta-analysis. Figure S3. Network plots of eligible comparisons for secondary outcomes: clinical pregnancy rate. a. Live birth; b. Embryo implantation; c. Chemical pregnancy; d. Miscarriage. Figure S4. Forest plot of the embryo implantation in direct pair-wise meta-analysis. Figure S5. Forest plot of the chemical pregnancy in direct pair-wise meta-analysis. Figure S6. Forest plot of the miscarriage in direct pair-wise meta-analysis. Figure S7. Funnel plot of the pregnancy outcomes. Figure S8. Subgroup analysis of forest plot of the clinical pregnancy in the direct pair-wise meta-analysis by English researches. Figure S9. Subgroup analysis of forest plot of the clinical pregnancy in the direct pair-wise meta-analysis by Chinese researches. Supplemental Table S1. Characteristics of studies included in meta-analyses. Supplemental Table S2. Risk of bias assessment of the other prospective studies. Supplemental Table S3. Network meta-analysis for live birth comparing diverse uterine infusion strategies. Supplemental Table S4. Network meta-analysis for implantation comparing diverse uterine infusion strategies. Supplemental Table S5. Network meta-analysis for chemical pregnancy comparing diverse uterine infusion strategies. Supplemental Table S6. Network meta-analysis for miscarriage comparing diverse uterine infusion strategies. Supplemental Table S7. Subgroup analysis of network meta-analysis for clinical pregnancy by English researches. Supplemental Table S8. Subgroup analysis of network meta-analysis for clinical pregnancy by Chinese researches. [file 12958_2024_1221_MOESM1_ESM.zip › Table S7 Sensitivity analysis-English.docx]

**Table S7** Subgroup analysis of Network meta-analysis for clinical pregnancy by English researches.

| **Groups/pregnant outcomes** | **GCSF** | **HCG** | **PBMC** | **PRP** | **PRP+G-CSFsc** | **Placebo** |
| --- | --- | --- | --- | --- | --- | --- |
| **Control** | 3.16 (1.77, 5.66) | 2.20 (1.28, 3.73) | 3.37 (2.12, 5.84) | 2.88 (1.90, 4.45) | 1.33 (0.34, 5.45) | 1.62 (0.96, 2.79) |
| **GCSF** |  | 0.69 (0.38, 1.24) | 1.06 (0.54, 2.26) | 0.91 (0.45, 1.84) | 0.42 (0.09, 1.96) | 0.51 (0.31, 0.86) |
| **HCG** |  |  | 1.54 (0.83, 3.08) | 1.32 (0.68, 2.60) | 0.61 (0.14, 2.77) | 0.74 (0.43, 1.27) |
| **PBMC** |  |  |  | 0.86 (0.44, 1.57) | 0.39 (0.09, 1.76) | 0.48 (0.24, 0.89) |
| **PRP** |  |  |  |  | 0.46 (0.11, 2.03) | 0.56 (0.29, 1.08) |
| **PRP+G-CSFsc** |  |  |  |  |  | 1.22 (0.27, 5.32) |
