# Supplementary material for: Uterine infusion strategies for infertile patients with recurrent implantation failure: a systematic review and network meta-analysis
Source: Reprod Biol Endocrinol. 2024 Apr 16;22:44. doi: 10.1186/s12958-024-01221-x (PMC11020641; doi:10.1186/s12958-024-01221-x)
Supplement: Supplementary file 1 — Additional file 1: Figure S1. Risk of bias assessment. a. Risk of bias summary; b. Risk of bias graph. Figure S2. Forest plot of the live birth in direct pair-wise meta-analysis. Figure S3. Network plots of eligible comparisons for secondary outcomes: clinical pregnancy rate. a. Live birth; b. Embryo implantation; c. Chemical pregnancy; d. Miscarriage. Figure S4. Forest plot of the embryo implantation in direct pair-wise meta-analysis. Figure S5. Forest plot of the chemical pregnancy in direct pair-wise meta-analysis. Figure S6. Forest plot of the miscarriage in direct pair-wise meta-analysis. Figure S7. Funnel plot of the pregnancy outcomes. Figure S8. Subgroup analysis of forest plot of the clinical pregnancy in the direct pair-wise meta-analysis by English researches. Figure S9. Subgroup analysis of forest plot of the clinical pregnancy in the direct pair-wise meta-analysis by Chinese researches. Supplemental Table S1. Characteristics of studies included in meta-analyses. Supplemental Table S2. Risk of bias assessment of the other prospective studies. Supplemental Table S3. Network meta-analysis for live birth comparing diverse uterine infusion strategies. Supplemental Table S4. Network meta-analysis for implantation comparing diverse uterine infusion strategies. Supplemental Table S5. Network meta-analysis for chemical pregnancy comparing diverse uterine infusion strategies. Supplemental Table S6. Network meta-analysis for miscarriage comparing diverse uterine infusion strategies. Supplemental Table S7. Subgroup analysis of network meta-analysis for clinical pregnancy by English researches. Supplemental Table S8. Subgroup analysis of network meta-analysis for clinical pregnancy by Chinese researches. [file 12958_2024_1221_MOESM1_ESM.zip › Table S8 Sensitivity analysis-chinese.docx]

**Table S8** Subgroup analysis of Network meta-analysis for clinical pregnancy by Chinese researches.

| **Groups/pregnant outcomes** | **DEX** | **ECS** | **G-CSF** | **G-CSF+AXaIUsc** | **GH** | **HCG** | **PBMC** | **PRP** | **PRP+G-CSF** | **Placebo** |
| --- | --- | --- | --- | --- | --- | --- | --- | --- | --- | --- |
| **Control** | 2.61 (1.25, 5.56) | 1.31 (0.67, 2.63) | 2.19 (1.54, 3.23) | 3.34 (1.24, 7.78) | 2.41 (1.02, 5.72) | 2.67 (1.80, 3.96) | 3.13 (2.27, 4.41) | 3.52 (2.17, 6.08) | 3.59 (1.30, 10.68) | 1.65 (1.14, 2.49) |
| **DEX** |  | 0.51 (0.18, 1.35) | 0.84 (0.36, 1.90) | 1.25 (0.36, 3.91) | 0.94 (0.31, 2.83) | 1.03 (0.42, 2.27) | 1.21 (0.54, 2.60) | 1.36 (0.55, 3.27) | 1.43 (0.37, 5.02) | 0.65 (0.27, 1.45) |
| **ECS** |  |  | 1.69 (0.75, 3.50) | 2.54 (0.75, 6.97) | 1.88 (0.63, 5.36) | 2.02 (0.92, 4.31) | 2.40 (1.12, 5.10) | 2.67 (1.17, 6.16) | 2.74 (0.79, 9.20) | 1.29 (0.57, 2.73) |
| **G-CSF** |  |  |  | 1.51 (0.59, 3.48) | 1.11 (0.48, 2.72) | 1.19 (0.79, 1.85) | 1.41 (0.96, 2.21) | 1.60 (0.84, 3.01) | 1.65 (0.56, 4.79) | 0.76 (0.51, 1.17) |
| **G-CSF+AXaIUsc** |  |  |  |  | 0.72 (0.24, 2.81) | 0.78 (0.33, 2.23) | 0.94 (0.39, 2.56) | 1.06 (0.40, 3.22) | 1.14 (0.29, 4.08) | 0.50 (0.21, 1.39) |
| **GH** |  |  |  |  |  | 1.07 (0.43, 2.65) | 1.28 (0.53, 3.32) | 1.42 (0.53, 3.83) | 1.45 (0.37, 6.17) | 0.68 (0.27, 1.63) |
| **HCG** |  |  |  |  |  |  | 1.18 (0.78, 1.84) | 1.31 (0.71, 2.49) | 1.37 (0.45, 4.19) | 0.63 (0.43, 0.92) |
| **PBMC** |  |  |  |  |  |  |  | 1.13 (0.61, 2.05) | 1.15 (0.39, 3.36) | 0.54 (0.35, 0.76) |
| **PRP** |  |  |  |  |  |  |  |  | 1.03 (0.33, 3.52) | 0.48 (0.25, 0.94) |
| **PRP+G-CSF** |  |  |  |  |  |  |  |  |  | 0.46 (0.16, 1.37) |
