# Supplementary figures and images for: Uterine infusion strategies for infertile patients with recurrent implantation failure: a systematic review and network meta-analysis
Source: Reprod Biol Endocrinol. 2024 Apr 16;22:44. doi: 10.1186/s12958-024-01221-x (PMC11020641; doi:10.1186/s12958-024-01221-x)

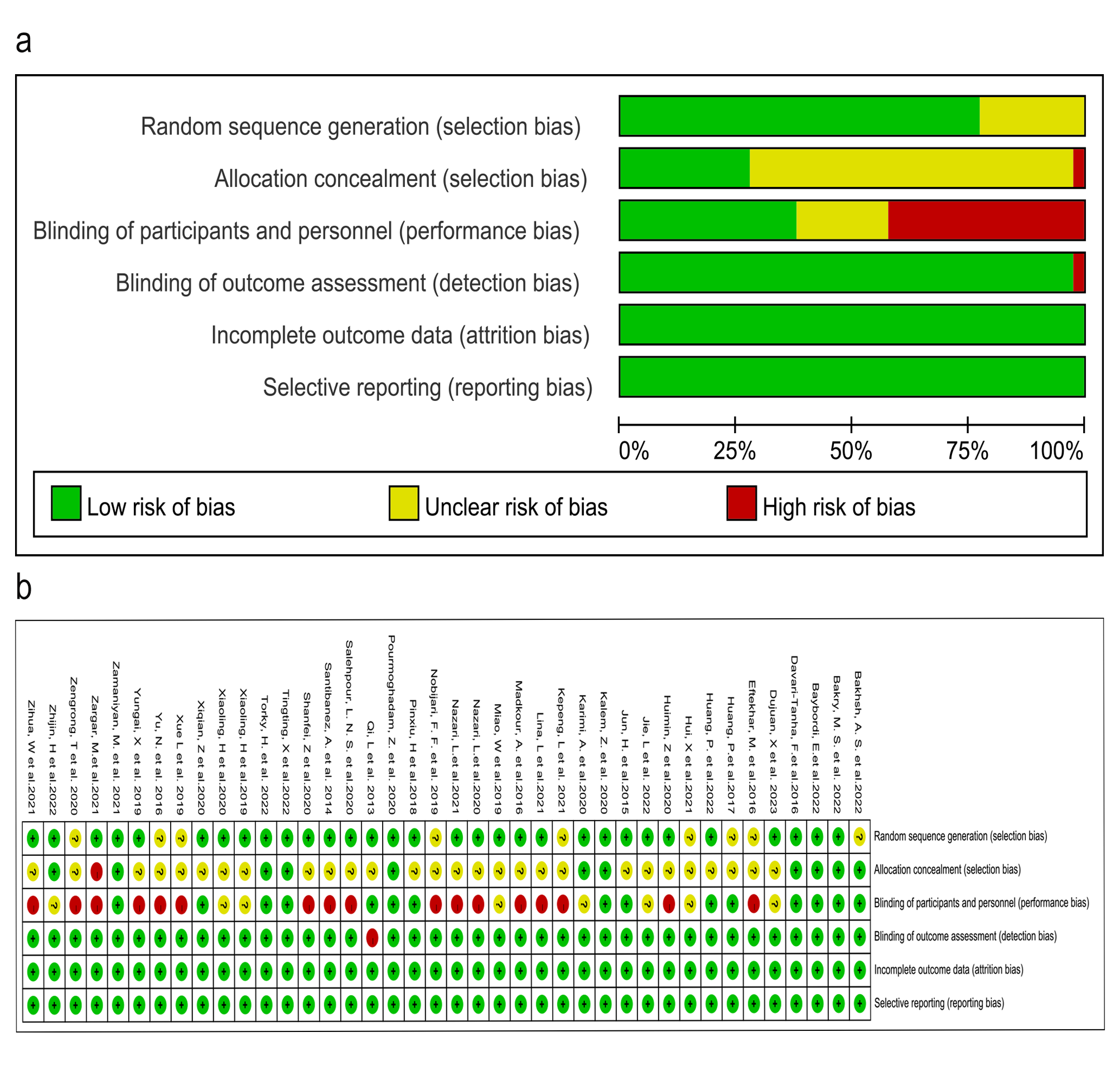

Supplement: Supplementary file 1 — Additional file 1: Figure S1. Risk of bias assessment. a. Risk of bias summary; b. Risk of bias graph. Figure S2. Forest plot of the live birth in direct pair-wise meta-analysis. Figure S3. Network plots of eligible comparisons for secondary outcomes: clinical pregnancy rate. a. Live birth; b. Embryo implantation; c. Chemical pregnancy; d. Miscarriage. Figure S4. Forest plot of the embryo implantation in direct pair-wise meta-analysis. Figure S5. Forest plot of the chemical pregnancy in direct pair-wise meta-analysis. Figure S6. Forest plot of the miscarriage in direct pair-wise meta-analysis. Figure S7. Funnel plot of the pregnancy outcomes. Figure S8. Subgroup analysis of forest plot of the clinical pregnancy in the direct pair-wise meta-analysis by English researches. Figure S9. Subgroup analysis of forest plot of the clinical pregnancy in the direct pair-wise meta-analysis by Chinese researches. Supplemental Table S1. Characteristics of studies included in meta-analyses. Supplemental Table S2. Risk of bias assessment of the other prospective studies. Supplemental Table S3. Network meta-analysis for live birth comparing diverse uterine infusion strategies. Supplemental Table S4. Network meta-analysis for implantation comparing diverse uterine infusion strategies. Supplemental Table S5. Network meta-analysis for chemical pregnancy comparing diverse uterine infusion strategies. Supplemental Table S6. Network meta-analysis for miscarriage comparing diverse uterine infusion strategies. Supplemental Table S7. Subgroup analysis of network meta-analysis for clinical pregnancy by English researches. Supplemental Table S8. Subgroup analysis of network meta-analysis for clinical pregnancy by Chinese researches. [file 12958_2024_1221_MOESM1_ESM.zip › Figure S1.tif]

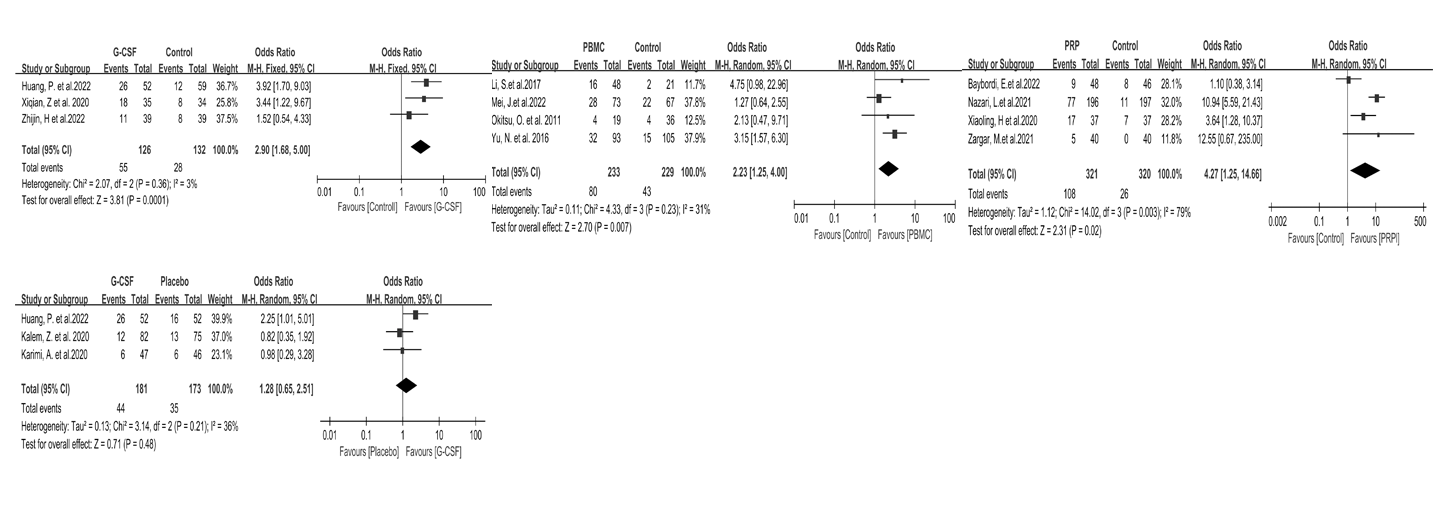

Supplement: Supplementary file 1 — Additional file 1: Figure S1. Risk of bias assessment. a. Risk of bias summary; b. Risk of bias graph. Figure S2. Forest plot of the live birth in direct pair-wise meta-analysis. Figure S3. Network plots of eligible comparisons for secondary outcomes: clinical pregnancy rate. a. Live birth; b. Embryo implantation; c. Chemical pregnancy; d. Miscarriage. Figure S4. Forest plot of the embryo implantation in direct pair-wise meta-analysis. Figure S5. Forest plot of the chemical pregnancy in direct pair-wise meta-analysis. Figure S6. Forest plot of the miscarriage in direct pair-wise meta-analysis. Figure S7. Funnel plot of the pregnancy outcomes. Figure S8. Subgroup analysis of forest plot of the clinical pregnancy in the direct pair-wise meta-analysis by English researches. Figure S9. Subgroup analysis of forest plot of the clinical pregnancy in the direct pair-wise meta-analysis by Chinese researches. Supplemental Table S1. Characteristics of studies included in meta-analyses. Supplemental Table S2. Risk of bias assessment of the other prospective studies. Supplemental Table S3. Network meta-analysis for live birth comparing diverse uterine infusion strategies. Supplemental Table S4. Network meta-analysis for implantation comparing diverse uterine infusion strategies. Supplemental Table S5. Network meta-analysis for chemical pregnancy comparing diverse uterine infusion strategies. Supplemental Table S6. Network meta-analysis for miscarriage comparing diverse uterine infusion strategies. Supplemental Table S7. Subgroup analysis of network meta-analysis for clinical pregnancy by English researches. Supplemental Table S8. Subgroup analysis of network meta-analysis for clinical pregnancy by Chinese researches. [file 12958_2024_1221_MOESM1_ESM.zip › Figure S2 livebirth.tif]

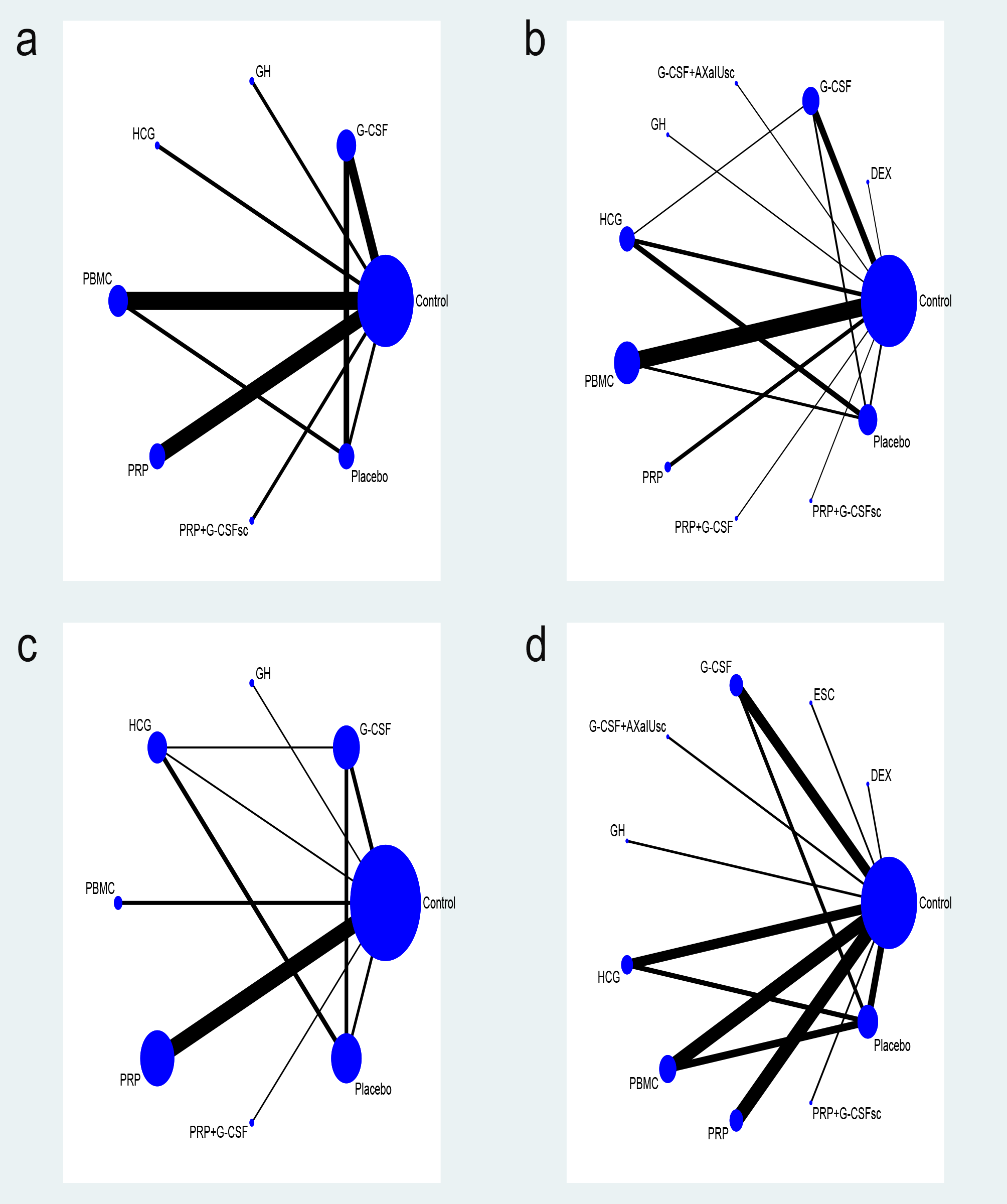

Supplement: Supplementary file 1 — Additional file 1: Figure S1. Risk of bias assessment. a. Risk of bias summary; b. Risk of bias graph. Figure S2. Forest plot of the live birth in direct pair-wise meta-analysis. Figure S3. Network plots of eligible comparisons for secondary outcomes: clinical pregnancy rate. a. Live birth; b. Embryo implantation; c. Chemical pregnancy; d. Miscarriage. Figure S4. Forest plot of the embryo implantation in direct pair-wise meta-analysis. Figure S5. Forest plot of the chemical pregnancy in direct pair-wise meta-analysis. Figure S6. Forest plot of the miscarriage in direct pair-wise meta-analysis. Figure S7. Funnel plot of the pregnancy outcomes. Figure S8. Subgroup analysis of forest plot of the clinical pregnancy in the direct pair-wise meta-analysis by English researches. Figure S9. Subgroup analysis of forest plot of the clinical pregnancy in the direct pair-wise meta-analysis by Chinese researches. Supplemental Table S1. Characteristics of studies included in meta-analyses. Supplemental Table S2. Risk of bias assessment of the other prospective studies. Supplemental Table S3. Network meta-analysis for live birth comparing diverse uterine infusion strategies. Supplemental Table S4. Network meta-analysis for implantation comparing diverse uterine infusion strategies. Supplemental Table S5. Network meta-analysis for chemical pregnancy comparing diverse uterine infusion strategies. Supplemental Table S6. Network meta-analysis for miscarriage comparing diverse uterine infusion strategies. Supplemental Table S7. Subgroup analysis of network meta-analysis for clinical pregnancy by English researches. Supplemental Table S8. Subgroup analysis of network meta-analysis for clinical pregnancy by Chinese researches. [file 12958_2024_1221_MOESM1_ESM.zip › Figure S3.tif]

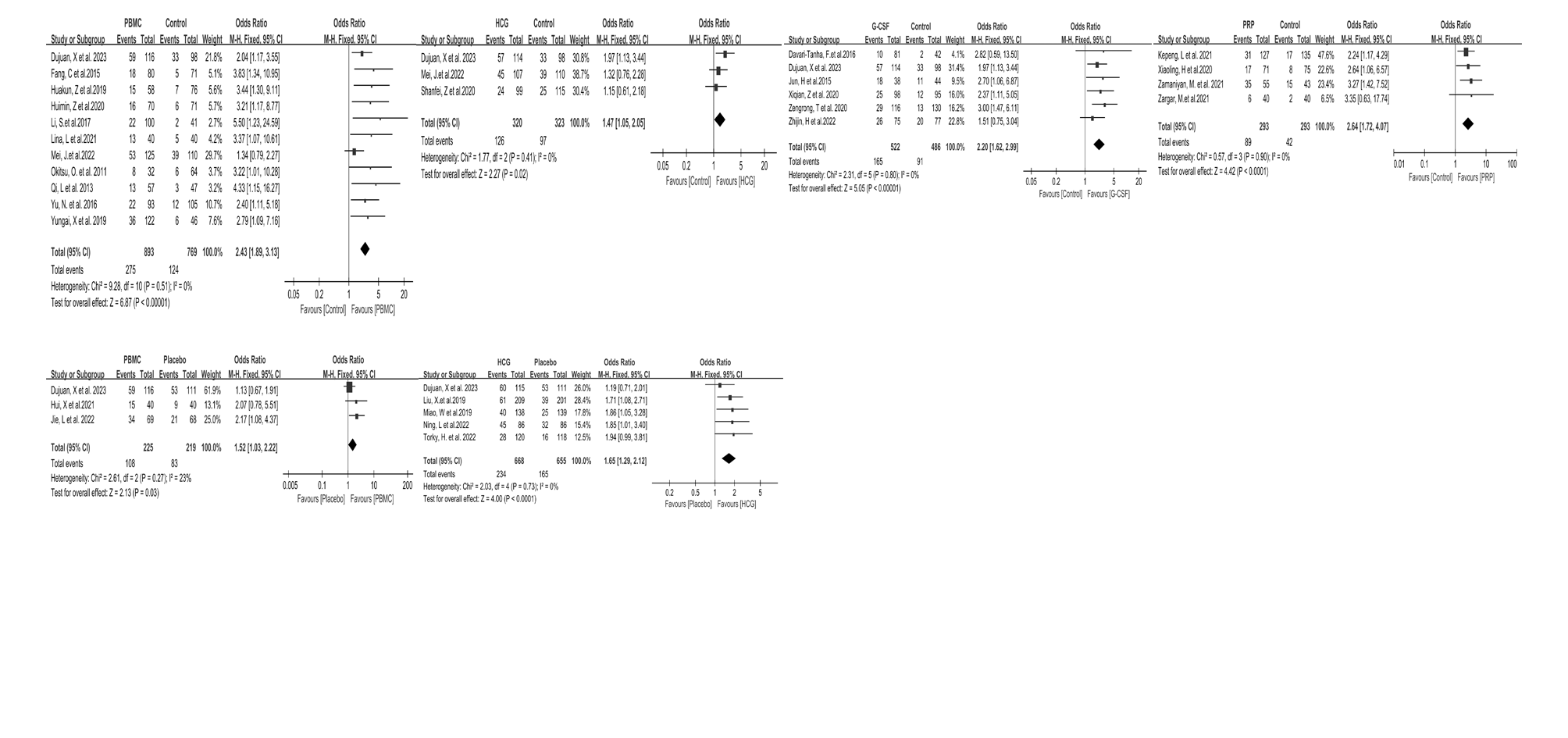

Supplement: Supplementary file 1 — Additional file 1: Figure S1. Risk of bias assessment. a. Risk of bias summary; b. Risk of bias graph. Figure S2. Forest plot of the live birth in direct pair-wise meta-analysis. Figure S3. Network plots of eligible comparisons for secondary outcomes: clinical pregnancy rate. a. Live birth; b. Embryo implantation; c. Chemical pregnancy; d. Miscarriage. Figure S4. Forest plot of the embryo implantation in direct pair-wise meta-analysis. Figure S5. Forest plot of the chemical pregnancy in direct pair-wise meta-analysis. Figure S6. Forest plot of the miscarriage in direct pair-wise meta-analysis. Figure S7. Funnel plot of the pregnancy outcomes. Figure S8. Subgroup analysis of forest plot of the clinical pregnancy in the direct pair-wise meta-analysis by English researches. Figure S9. Subgroup analysis of forest plot of the clinical pregnancy in the direct pair-wise meta-analysis by Chinese researches. Supplemental Table S1. Characteristics of studies included in meta-analyses. Supplemental Table S2. Risk of bias assessment of the other prospective studies. Supplemental Table S3. Network meta-analysis for live birth comparing diverse uterine infusion strategies. Supplemental Table S4. Network meta-analysis for implantation comparing diverse uterine infusion strategies. Supplemental Table S5. Network meta-analysis for chemical pregnancy comparing diverse uterine infusion strategies. Supplemental Table S6. Network meta-analysis for miscarriage comparing diverse uterine infusion strategies. Supplemental Table S7. Subgroup analysis of network meta-analysis for clinical pregnancy by English researches. Supplemental Table S8. Subgroup analysis of network meta-analysis for clinical pregnancy by Chinese researches. [file 12958_2024_1221_MOESM1_ESM.zip › Figure S4 implantation.tif]

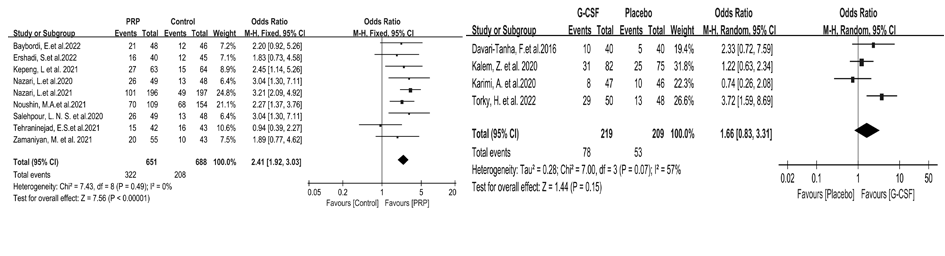

Supplement: Supplementary file 1 — Additional file 1: Figure S1. Risk of bias assessment. a. Risk of bias summary; b. Risk of bias graph. Figure S2. Forest plot of the live birth in direct pair-wise meta-analysis. Figure S3. Network plots of eligible comparisons for secondary outcomes: clinical pregnancy rate. a. Live birth; b. Embryo implantation; c. Chemical pregnancy; d. Miscarriage. Figure S4. Forest plot of the embryo implantation in direct pair-wise meta-analysis. Figure S5. Forest plot of the chemical pregnancy in direct pair-wise meta-analysis. Figure S6. Forest plot of the miscarriage in direct pair-wise meta-analysis. Figure S7. Funnel plot of the pregnancy outcomes. Figure S8. Subgroup analysis of forest plot of the clinical pregnancy in the direct pair-wise meta-analysis by English researches. Figure S9. Subgroup analysis of forest plot of the clinical pregnancy in the direct pair-wise meta-analysis by Chinese researches. Supplemental Table S1. Characteristics of studies included in meta-analyses. Supplemental Table S2. Risk of bias assessment of the other prospective studies. Supplemental Table S3. Network meta-analysis for live birth comparing diverse uterine infusion strategies. Supplemental Table S4. Network meta-analysis for implantation comparing diverse uterine infusion strategies. Supplemental Table S5. Network meta-analysis for chemical pregnancy comparing diverse uterine infusion strategies. Supplemental Table S6. Network meta-analysis for miscarriage comparing diverse uterine infusion strategies. Supplemental Table S7. Subgroup analysis of network meta-analysis for clinical pregnancy by English researches. Supplemental Table S8. Subgroup analysis of network meta-analysis for clinical pregnancy by Chinese researches. [file 12958_2024_1221_MOESM1_ESM.zip › Figure S5 chemical pregnancy.tif]

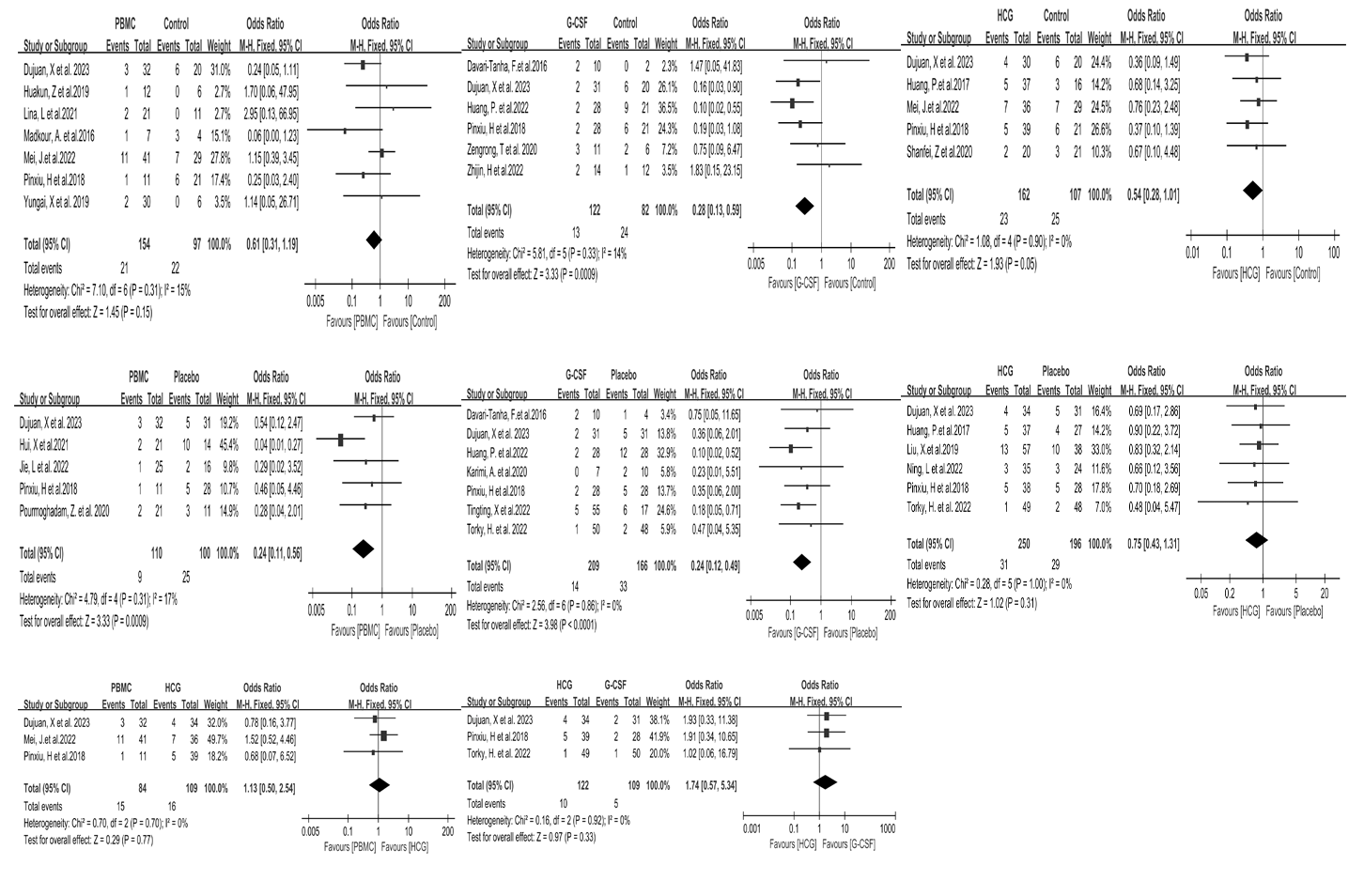

Supplement: Supplementary file 1 — Additional file 1: Figure S1. Risk of bias assessment. a. Risk of bias summary; b. Risk of bias graph. Figure S2. Forest plot of the live birth in direct pair-wise meta-analysis. Figure S3. Network plots of eligible comparisons for secondary outcomes: clinical pregnancy rate. a. Live birth; b. Embryo implantation; c. Chemical pregnancy; d. Miscarriage. Figure S4. Forest plot of the embryo implantation in direct pair-wise meta-analysis. Figure S5. Forest plot of the chemical pregnancy in direct pair-wise meta-analysis. Figure S6. Forest plot of the miscarriage in direct pair-wise meta-analysis. Figure S7. Funnel plot of the pregnancy outcomes. Figure S8. Subgroup analysis of forest plot of the clinical pregnancy in the direct pair-wise meta-analysis by English researches. Figure S9. Subgroup analysis of forest plot of the clinical pregnancy in the direct pair-wise meta-analysis by Chinese researches. Supplemental Table S1. Characteristics of studies included in meta-analyses. Supplemental Table S2. Risk of bias assessment of the other prospective studies. Supplemental Table S3. Network meta-analysis for live birth comparing diverse uterine infusion strategies. Supplemental Table S4. Network meta-analysis for implantation comparing diverse uterine infusion strategies. Supplemental Table S5. Network meta-analysis for chemical pregnancy comparing diverse uterine infusion strategies. Supplemental Table S6. Network meta-analysis for miscarriage comparing diverse uterine infusion strategies. Supplemental Table S7. Subgroup analysis of network meta-analysis for clinical pregnancy by English researches. Supplemental Table S8. Subgroup analysis of network meta-analysis for clinical pregnancy by Chinese researches. [file 12958_2024_1221_MOESM1_ESM.zip › Figure S6 miscarriage.tif]

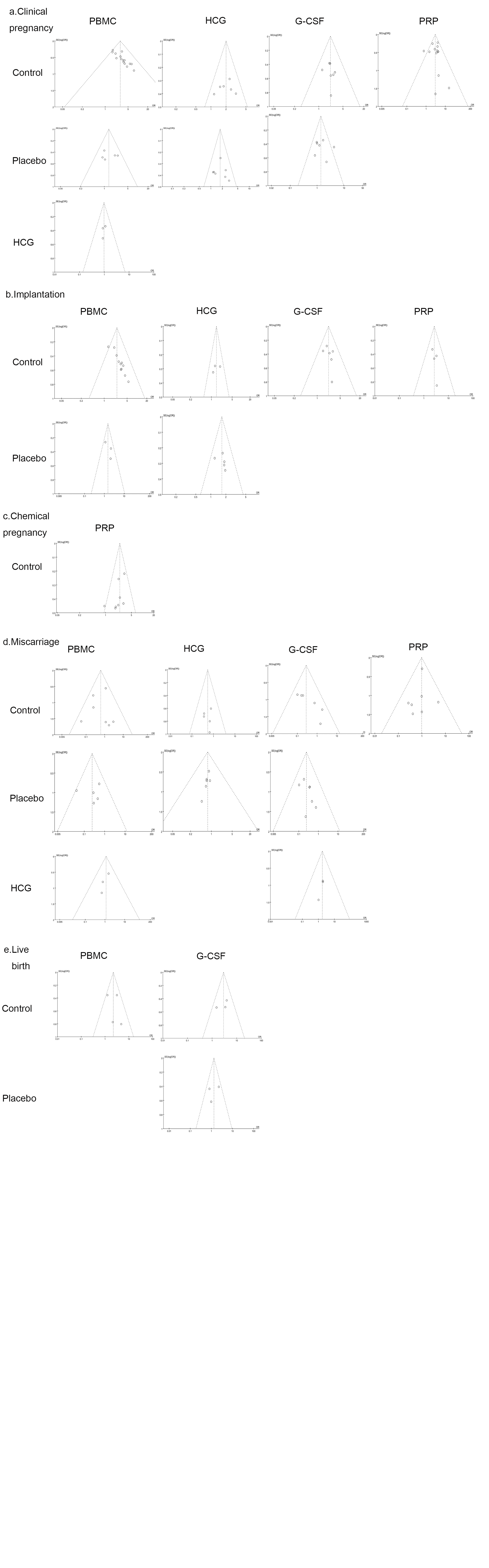

Supplement: Supplementary file 1 — Additional file 1: Figure S1. Risk of bias assessment. a. Risk of bias summary; b. Risk of bias graph. Figure S2. Forest plot of the live birth in direct pair-wise meta-analysis. Figure S3. Network plots of eligible comparisons for secondary outcomes: clinical pregnancy rate. a. Live birth; b. Embryo implantation; c. Chemical pregnancy; d. Miscarriage. Figure S4. Forest plot of the embryo implantation in direct pair-wise meta-analysis. Figure S5. Forest plot of the chemical pregnancy in direct pair-wise meta-analysis. Figure S6. Forest plot of the miscarriage in direct pair-wise meta-analysis. Figure S7. Funnel plot of the pregnancy outcomes. Figure S8. Subgroup analysis of forest plot of the clinical pregnancy in the direct pair-wise meta-analysis by English researches. Figure S9. Subgroup analysis of forest plot of the clinical pregnancy in the direct pair-wise meta-analysis by Chinese researches. Supplemental Table S1. Characteristics of studies included in meta-analyses. Supplemental Table S2. Risk of bias assessment of the other prospective studies. Supplemental Table S3. Network meta-analysis for live birth comparing diverse uterine infusion strategies. Supplemental Table S4. Network meta-analysis for implantation comparing diverse uterine infusion strategies. Supplemental Table S5. Network meta-analysis for chemical pregnancy comparing diverse uterine infusion strategies. Supplemental Table S6. Network meta-analysis for miscarriage comparing diverse uterine infusion strategies. Supplemental Table S7. Subgroup analysis of network meta-analysis for clinical pregnancy by English researches. Supplemental Table S8. Subgroup analysis of network meta-analysis for clinical pregnancy by Chinese researches. [file 12958_2024_1221_MOESM1_ESM.zip › Figure S7.tif]

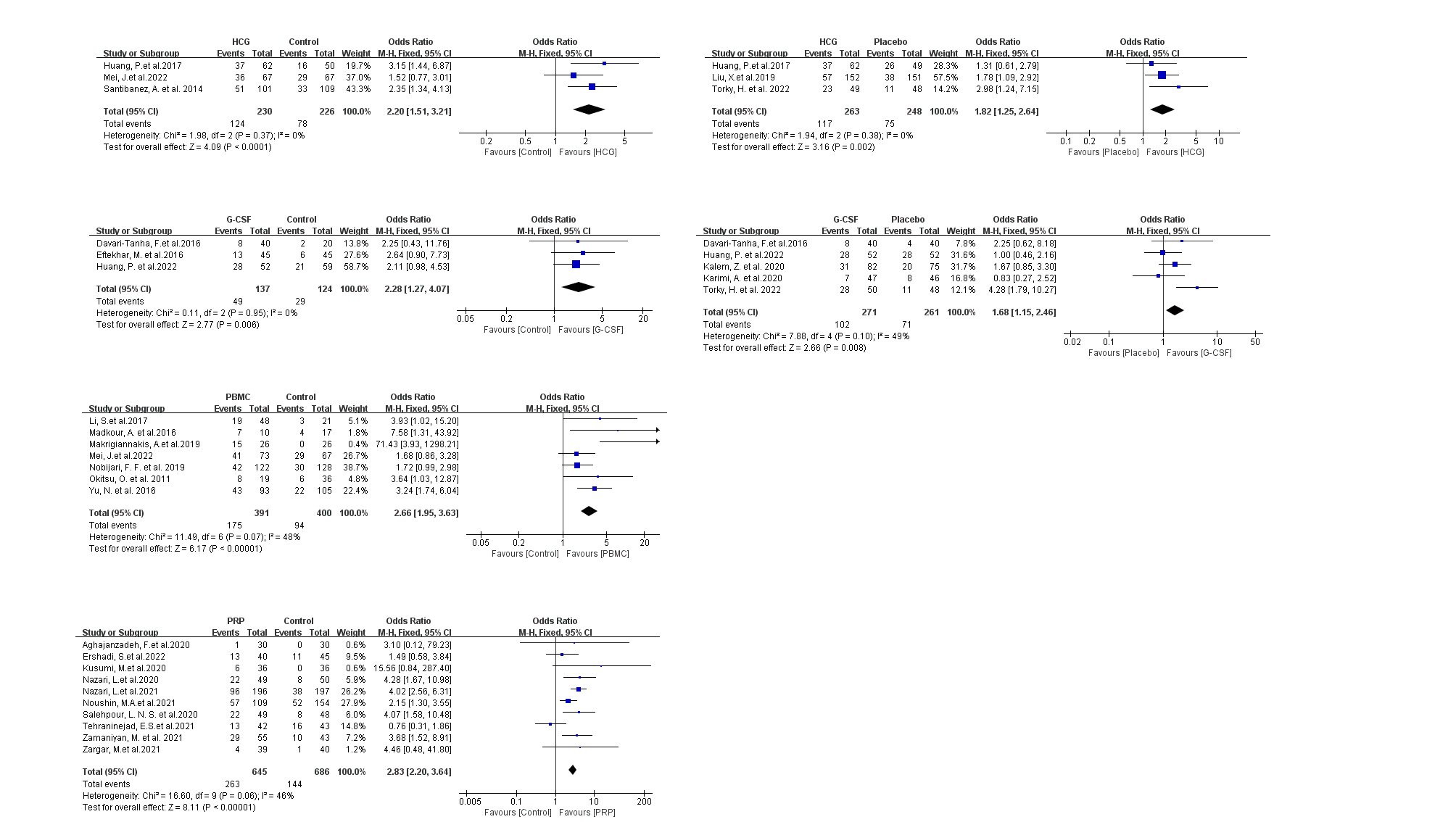

Supplement: Supplementary file 1 — Additional file 1: Figure S1. Risk of bias assessment. a. Risk of bias summary; b. Risk of bias graph. Figure S2. Forest plot of the live birth in direct pair-wise meta-analysis. Figure S3. Network plots of eligible comparisons for secondary outcomes: clinical pregnancy rate. a. Live birth; b. Embryo implantation; c. Chemical pregnancy; d. Miscarriage. Figure S4. Forest plot of the embryo implantation in direct pair-wise meta-analysis. Figure S5. Forest plot of the chemical pregnancy in direct pair-wise meta-analysis. Figure S6. Forest plot of the miscarriage in direct pair-wise meta-analysis. Figure S7. Funnel plot of the pregnancy outcomes. Figure S8. Subgroup analysis of forest plot of the clinical pregnancy in the direct pair-wise meta-analysis by English researches. Figure S9. Subgroup analysis of forest plot of the clinical pregnancy in the direct pair-wise meta-analysis by Chinese researches. Supplemental Table S1. Characteristics of studies included in meta-analyses. Supplemental Table S2. Risk of bias assessment of the other prospective studies. Supplemental Table S3. Network meta-analysis for live birth comparing diverse uterine infusion strategies. Supplemental Table S4. Network meta-analysis for implantation comparing diverse uterine infusion strategies. Supplemental Table S5. Network meta-analysis for chemical pregnancy comparing diverse uterine infusion strategies. Supplemental Table S6. Network meta-analysis for miscarriage comparing diverse uterine infusion strategies. Supplemental Table S7. Subgroup analysis of network meta-analysis for clinical pregnancy by English researches. Supplemental Table S8. Subgroup analysis of network meta-analysis for clinical pregnancy by Chinese researches. [file 12958_2024_1221_MOESM1_ESM.zip › Figure S8.tif]

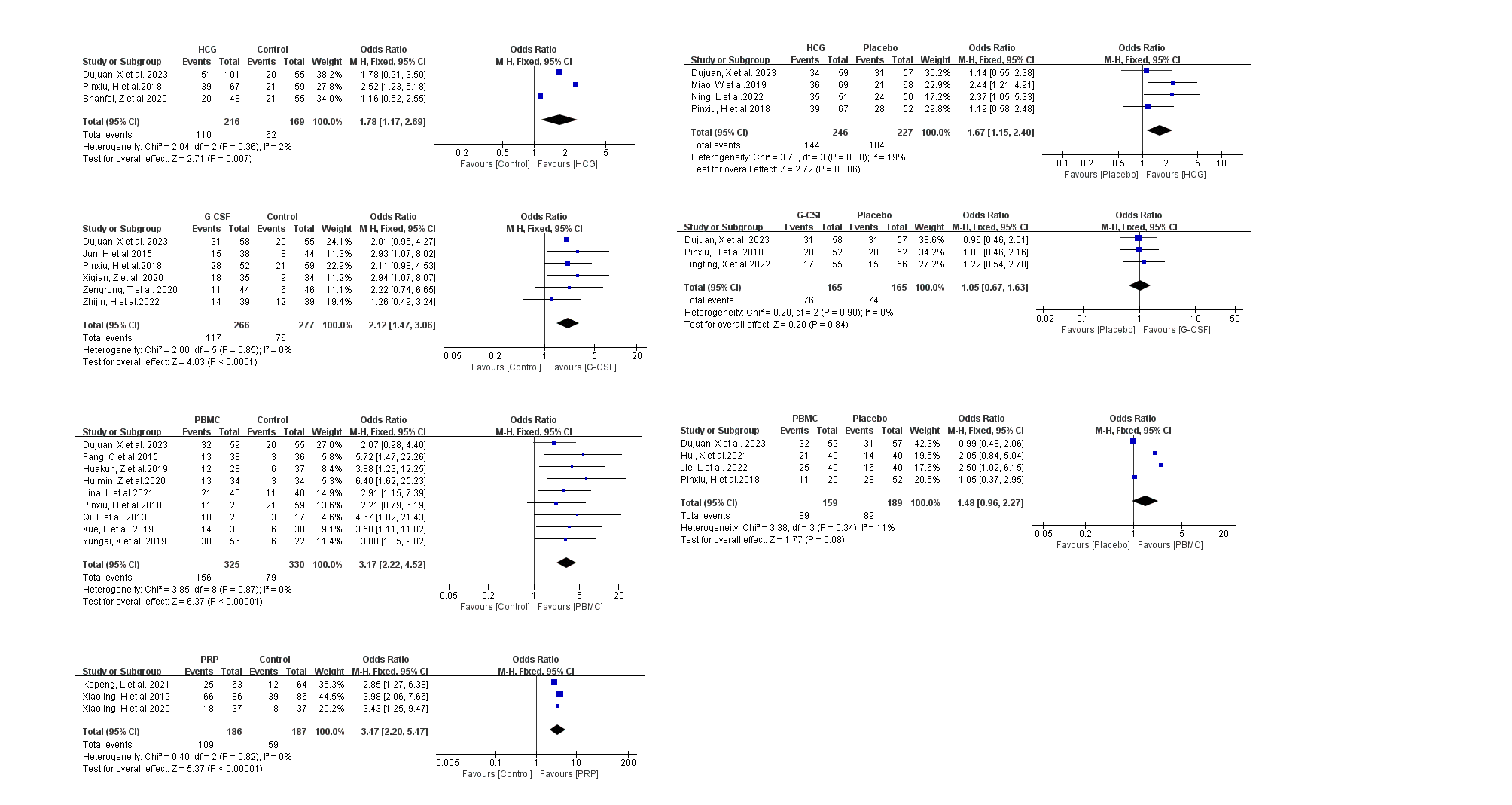

Supplement: Supplementary file 1 — Additional file 1: Figure S1. Risk of bias assessment. a. Risk of bias summary; b. Risk of bias graph. Figure S2. Forest plot of the live birth in direct pair-wise meta-analysis. Figure S3. Network plots of eligible comparisons for secondary outcomes: clinical pregnancy rate. a. Live birth; b. Embryo implantation; c. Chemical pregnancy; d. Miscarriage. Figure S4. Forest plot of the embryo implantation in direct pair-wise meta-analysis. Figure S5. Forest plot of the chemical pregnancy in direct pair-wise meta-analysis. Figure S6. Forest plot of the miscarriage in direct pair-wise meta-analysis. Figure S7. Funnel plot of the pregnancy outcomes. Figure S8. Subgroup analysis of forest plot of the clinical pregnancy in the direct pair-wise meta-analysis by English researches. Figure S9. Subgroup analysis of forest plot of the clinical pregnancy in the direct pair-wise meta-analysis by Chinese researches. Supplemental Table S1. Characteristics of studies included in meta-analyses. Supplemental Table S2. Risk of bias assessment of the other prospective studies. Supplemental Table S3. Network meta-analysis for live birth comparing diverse uterine infusion strategies. Supplemental Table S4. Network meta-analysis for implantation comparing diverse uterine infusion strategies. Supplemental Table S5. Network meta-analysis for chemical pregnancy comparing diverse uterine infusion strategies. Supplemental Table S6. Network meta-analysis for miscarriage comparing diverse uterine infusion strategies. Supplemental Table S7. Subgroup analysis of network meta-analysis for clinical pregnancy by English researches. Supplemental Table S8. Subgroup analysis of network meta-analysis for clinical pregnancy by Chinese researches. [file 12958_2024_1221_MOESM1_ESM.zip › Figure S9.tif]
